# Supplementary material for: In hot water: Uncertainties in projecting marine heatwaves impacts on seagrass meadows
Source: PLoS One. 2024 Nov 27;19(11):e0298853. doi: 10.1371/journal.pone.0298853 (PMC11602073; doi:10.1371/journal.pone.0298853)
Supplement: S1 Fig — A Bayesian Network (BN) is a class of graphical models representing the probabilistic relationships among a set of variables (or nodes) with directed arcs between them. These variables form a directed acyclic graph (DAG), and each node in the DAG has a state that varies depending on the states of other nodes [147]. Information about those states is transmitted throughout the DAG, and as a result, inferences may be made by adding new data or evidence to the network. While BNs are suitable approaches for inferring static processes, the Dynamic Bayesian Network (DBN), a BN extension in which nodes represent variables at specific time slices, may be used to model temporally varying processes in which the state of a variable may change over time [148]. When adding a dynamic component to a BN and creating a DBN, the characteristic processes of complex systems, including cumulative effects and feedback processes, can be captured. The DBN approach allows us to predict the resilience of a system given the temporal dynamics of the components of the ecosystem and their interactions with natural and anthropogenic stressors [101, 107]. The overall DBN network structure is depicted with ovals representing factors (or nodes) and arrows indicating causal parent-child relationships. In this structure, a parent node (e.g., Meadow Type) exerts an influence on a child node (e.g., Location Type). Conversely, the absence of a link between nodes signifies conditional independence. Rounded rectangles denote subnetworks [77]. Note the presence of complex interdependencies and feedback loops in the seagrass ecosystem. Node colors denote different categories: site condition (white), recovery (purple), resistance (green), environmental (blue), and population (yellow). The following symbology is used in this figure: A node with a check mark: A node is ticked when inference has been successfully executed. A curved arrow back onto itself: Denotes a link to the node’s next time slice (i.e., t + 1) [file pone.0298853.s001.pdf]

S1 Fig. Seagrass Dynamic Bayesian Network. A Bayesian Network (BN) is a class of graphical models representing the probabilistic relationships among a set of variables (or nodes) with directed arcs between them. These variables form a directed acyclic graph (DAG), and each node in the DAG has a state that varies depending on the states of other nodes [1]. Information about those states is transmitted throughout the DAG, and as a result, inferences may be made by adding new data or evidence to the network. While BNs are suitable approaches for inferring static processes, the Dynamic Bayesian Network (DBN), a BN extension in which nodes represent variables at specific time slices, may be used to model temporally varying processes in which the state of a variable may change over time [2]. When adding a dynamic component to a BN and creating a DBN, the characteristic processes of complex systems, including cumulative effects and feedback processes, can be captured. The DBN approach allows us to predict the resilience of a system given the temporal dynamics of the components of the ecosystem and their interactions with natural and anthropogenic stressors [3, 4].

The overall DBN network structure is depicted with ovals representing factors (or nodes) and arrows indicating causal parent-child relationships. In this structure, a parent node (e.g., Meadow Type) exerts an influence on a child node (e.g., Location Type). Conversely, the absence of a link between nodes signifies conditional independence. Rounded rectangles denote subnetworks [5]. Note the presence of complex interdependencies and feedback loops in the seagrass ecosystem. Node colors denote different categories: site condition (white), recovery (purple), resistance (green), environmental (blue), and population (yellow). The following symbology is used in this figure: A node with a check mark: A node is ticked when inference has been successfully executed. A curved arrow back onto itself: Denotes a link to the node's next time slice (i.e., $t+1$). A double-headed arrow: Indicates that arcs are heading both ways between two subnetworks. For example, a double-headed arrow between a node N and a subnetwork S means that there is at least one node in S that depends on N and that there is at least one node in S that influences N. An arrow labeled with a [1]: Indicates a connection to a subsequent time slice ($t + 1$).

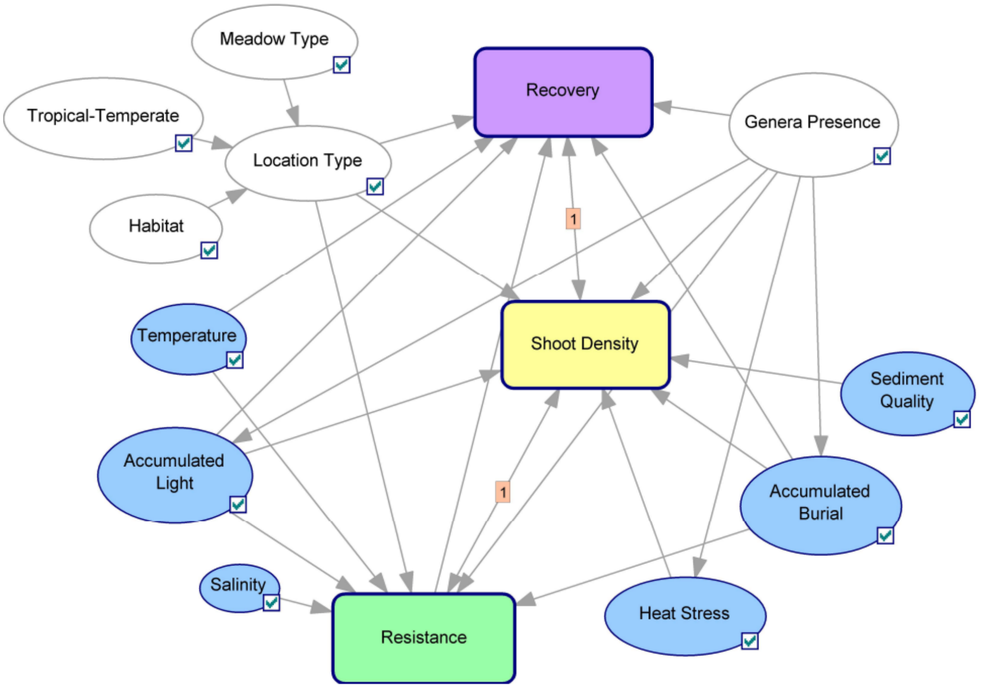

References

[1] Jensen FV, Nielsen TD. Bayesian networks and decision graphs. 2nd ed. Springer; 2007.

[2] Murphy K, Mian S. Modelling gene expression data using dynamic Bayesian networks. *Citeseer*. 1999.

[3] Wu PPY, Mengersen K, McMahon K, Kendrick GA, Chartrand K, York PH, et al. Timing anthropogenic stressors to mitigate their impact on marine ecosystem resilience. *Nature Communications*. 2017;8(1):1–11.

[4] Wu PPY, McMahon K, Rasheed MA, Kendrick GA, York PH, Chartrand K, et al. Managing seagrass resilience under cumulative dredging affecting light: Predicting risk using dynamic Bayesian networks. *Journal of Applied Ecology*. 2018;55(3):1339–1350.

[5] Johnson S, Mengersen K. Integrated Bayesian network framework for modeling complex ecological issues. *Integrated Environmental Assessment and Management*. 2012;8(3):480–490.
