# Supplementary material for: In hot water: Uncertainties in projecting marine heatwaves impacts on seagrass meadows
Source: PLoS One. 2024 Nov 27;19(11):e0298853. doi: 10.1371/journal.pone.0298853 (PMC11602073; doi:10.1371/journal.pone.0298853)

S3 Fig. Annual projections for low shoot density ratio of *Z. muelleri* in Gladstone, Australia, depicting different socio-economic pathway scenarios from 2030 to 2100. Each colour corresponds to a distinct scenario: red (SSP1-1.9), green (SSP1-2.6), blue (SSP3-7.0), and purple (SSP5-8.5). The lines represent key statistical measures: solid lines for the average, dashed lines for the 95th percentile (upper) and 5th percentile (lower), a grey area for the 75th percentile (upper) and 25th percentile (lower) derived from a dataset of 100 samples.

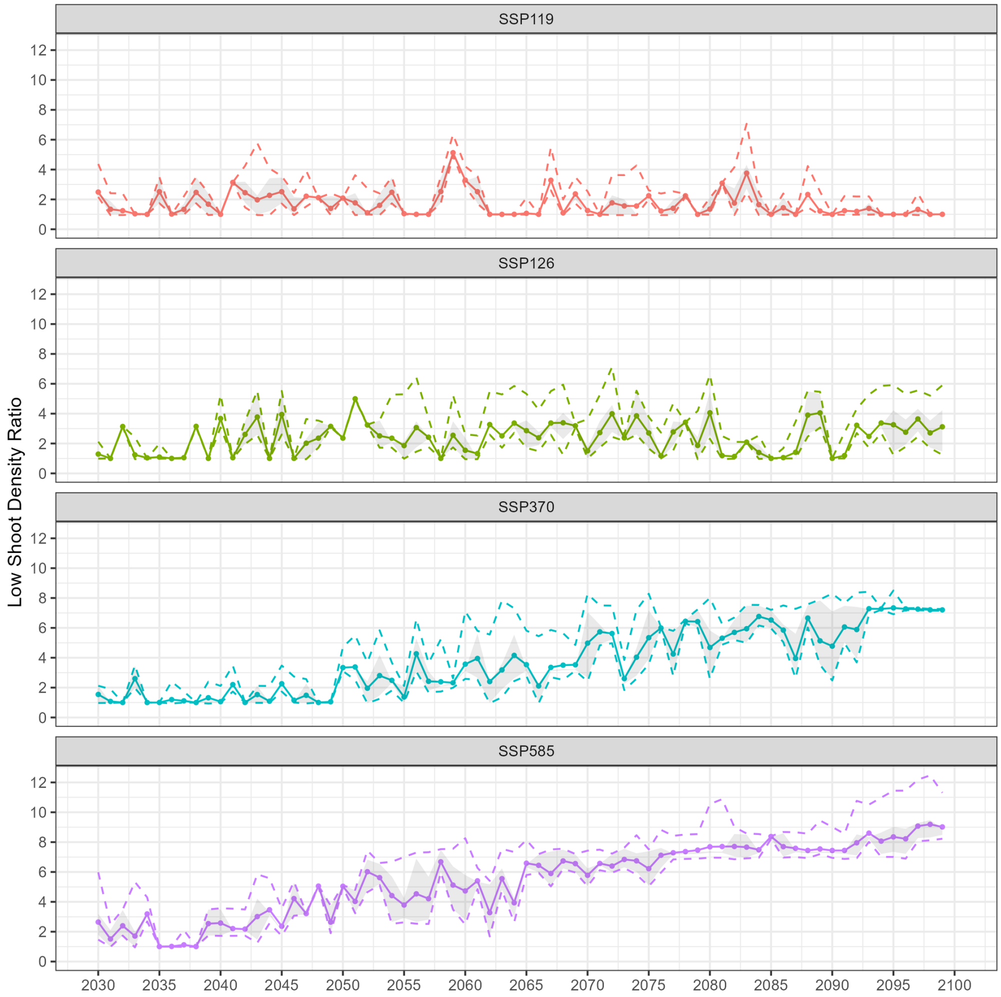

Supplement: S3 Fig — Each colour corresponds to a distinct scenario: red (SSP1-1.9), green (SSP1-2.6), blue (SSP3-7.0), and purple (SSP5-8.5). The lines represent key statistical measures: solid lines for the average, dashed lines for the 95th percentile (upper) and 5th percentile (lower), a grey area for the 75th percentile (upper) and 25th percentile (lower) derived from a dataset of 100 samples. (PDF) [file pone.0298853.s003.pdf]
