# Supplementary material for: In hot water: Uncertainties in projecting marine heatwaves impacts on seagrass meadows
Source: PLoS One. 2024 Nov 27;19(11):e0298853. doi: 10.1371/journal.pone.0298853 (PMC11602073; doi:10.1371/journal.pone.0298853)

S4 Fig. Recovery time. The estimation of recovery time $q(y)$ is calculated as $q(y) = y' - y$, where y is the first year when the high ratio $r_{\text{high}}(y)$ falls below 90% of the baseline. Then y' is the earliest subsequent year when the high ratio $r_{\text{high}}(y')$ returns to or exceeds 90% of the baseline.

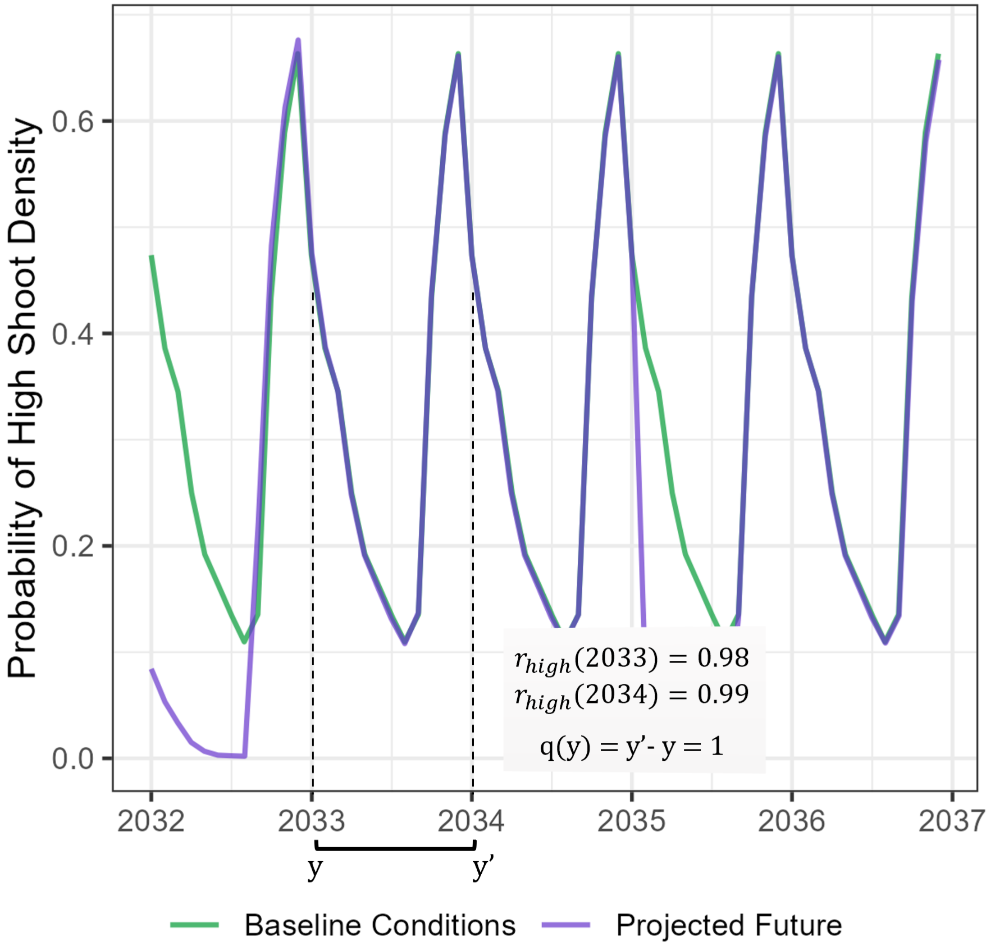

Supplement: S4 Fig — The estimation of recovery time q(y) is calculated as q(y) = y′ − y, where y is the first year when the high ratio rhigh(y) falls below 90% of the baseline. Then y′ is the earliest subsequent year when the high ratio rhigh(y′) returns to or exceeds 90% of the baseline. (PDF) [file pone.0298853.s004.pdf]
