# Supplementary material for: In hot water: Uncertainties in projecting marine heatwaves impacts on seagrass meadows
Source: PLoS One. 2024 Nov 27;19(11):e0298853. doi: 10.1371/journal.pone.0298853 (PMC11602073; doi:10.1371/journal.pone.0298853)

S5 Fig. Baseline return time for high shoot density ratios of *Z. muelleri* in Gladstone, Australia, depicting various socio-economic pathway scenarios from 2030 to 2100. Each color represents a distinct scenario, as follows: red (SSP1-1.9), green (SSP1-2.6), blue (SSP3-7.0), and purple (SSP5-8.5). The lollipop graphs illustrate the number of years since the last event where the high shoot density ratio was equal to or exceeded 0.8.

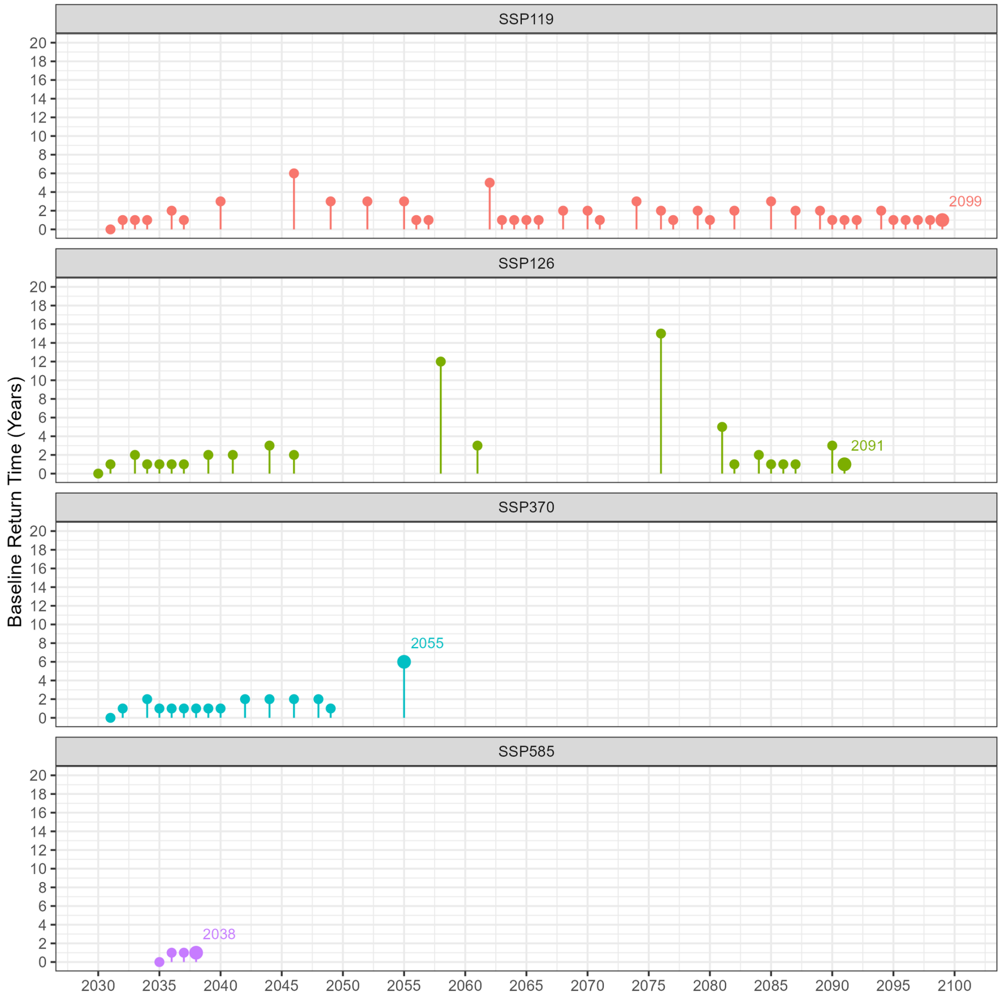

Supplement: S5 Fig — Each color represents a distinct scenario, as follows: red (SSP1-1.9), green (SSP1-2.6), blue (SSP3-7.0), and purple (SSP5-8.5). The lollipop graphs illustrate the number of years since the last event where the high shoot density ratio was equal to or exceeded 0.8. (PDF) [file pone.0298853.s005.pdf]
