# Supplementary material for: In hot water: Uncertainties in projecting marine heatwaves impacts on seagrass meadows
Source: PLoS One. 2024 Nov 27;19(11):e0298853. doi: 10.1371/journal.pone.0298853 (PMC11602073; doi:10.1371/journal.pone.0298853)

**S8 Fig. Predicted-state probabilities for high shoot density and the effect of heat stress on *Z. muelleri* in Gladstone, Australia, under the SSP1-2.6 scenario.** **(a)** The green line represents the baseline high shoot density, and the purple lines represent the projected high shoot density. **(b)** The green line illustrates the baseline effect of heat stress, while the red lines depict the projected impact of heat stress. No baseline is represented for heat stress, as its value is consistently zero. **(c)** The green line portrays the baseline temperature effect, and the orange lines indicate the projected sub-optimal temperatures.

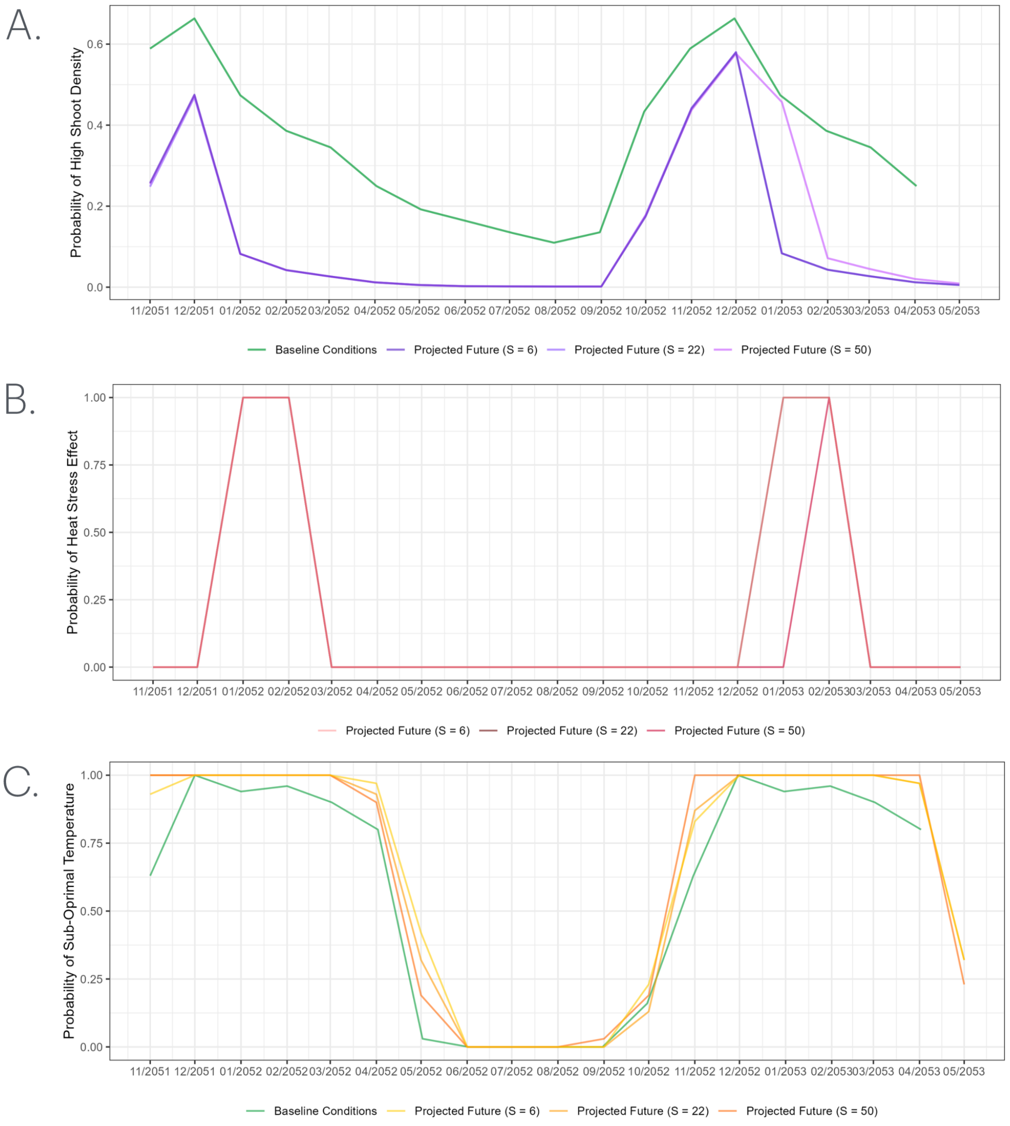

Supplement: S8 Fig — (a) The green line represents the baseline high shoot density, and the purple lines represent the projected high shoot density. (b) The green line illustrates the baseline effect of heat stress, while the red lines depict the projected impact of heat stress. No baseline is represented for heat stress, as its value is consistently zero. (c) The green line portrays the baseline temperature effect, and the orange lines indicate the projected sub-optimal temperatures. (PDF) [file pone.0298853.s008.pdf]
