# Supplementary material for: In hot water: Uncertainties in projecting marine heatwaves impacts on seagrass meadows
Source: PLoS One. 2024 Nov 27;19(11):e0298853. doi: 10.1371/journal.pone.0298853 (PMC11602073; doi:10.1371/journal.pone.0298853)
Supplement: S1 Table — Avg: denotes the average high shoot density ratio per decade. Q25: represents 25th percentile, marking the value below which 25% of the observations fall. Q95: stands for the 95th percentile indicating the value below which 95% of the observations are found. (PDF) [file pone.0298853.s009.pdf]

S1 Table. High Shoot Density Ratio Across Years for SSP1-1.9 Scenario: This table provides an analysis of the high shoot density states, measured annually within the SSP1-1.9 scenario. Avg: denotes the average high shoot density ratio per decade. **Q25:** represents 25th percentile, marking the value below which 25% of the observations fall. **Q95:** stands for the 95th percentile indicating the value below which 95% of the observations are found.

Scenario	Year	Average	Q5	Q25	Q75	Q95
SSP1-1.9	2030	0.4209	0.2115	0.4463	0.4509	0.4544
SSP1-1.9	2031	0.8602	0.3719	0.6696	1.0195	1.0674
SSP1-1.9	2032	0.8950	0.3749	0.9895	0.9940	1.0655
SSP1-1.9	2033	0.9823	0.9915	0.9931	0.9964	0.9994
SSP1-1.9	2034	0.9958	0.9901	0.9932	0.9983	1.0020
SSP1-1.9	2035	0.3608	0.2218	0.2228	0.4570	0.4604
SSP1-1.9	2036	0.9908	0.9830	0.9867	0.9954	0.9991
SSP1-1.9	2037	0.8150	0.3543	0.5150	0.9934	0.9968
SSP1-1.9	2038	0.3697	0.2181	0.2200	0.4551	0.4570
SSP1-1.9	2039	0.7331	0.3749	0.3793	1.0567	1.0664
SSP1-1.9	2040	0.9940	0.9890	0.9916	0.9965	0.9998
SSP1-1.9	2041	0.2187	0.2165	0.2180	0.2196	0.2207
SSP1-1.9	2042	0.3914	0.1805	0.2950	0.4487	0.4520
SSP1-1.9	2043	0.6508	0.1519	0.3714	0.9928	1.0642
SSP1-1.9	2044	0.5045	0.1976	0.2253	0.5503	1.0760
SSP1-1.9	2045	0.3670	0.2148	0.2193	0.4554	0.4595
SSP1-1.9	2046	0.8419	0.3686	0.5118	1.0237	1.0594
SSP1-1.9	2047	0.4379	0.2658	0.4540	0.4587	0.4625
SSP1-1.9	2048	0.4487	0.4436	0.4533	0.4583	0.4622
SSP1-1.9	2049	0.8312	0.3719	0.5138	1.0490	1.0646
SSP1-1.9	2050	0.4530	0.4497	0.4515	0.4544	0.4574
SSP1-1.9	2051	0.6670	0.2222	0.3763	1.0616	1.0699
SSP1-1.9	2052	0.9900	0.9254	0.9909	0.9966	0.9995
SSP1-1.9	2053	0.7469	0.3734	0.3761	1.0649	1.0701
SSP1-1.9	2054	0.3689	0.2200	0.2222	0.4598	0.4659
SSP1-1.9	2055	0.9706	0.9823	0.9864	0.9965	1.0013
SSP1-1.9	2056	0.9935	0.9872	0.9901	0.9963	1.0004
SSP1-1.9	2057	0.9941	0.9906	0.9924	0.9955	0.9985
SSP1-1.9	2058	0.3550	0.2168	0.2187	0.4542	0.4576
SSP1-1.9	2059	0.1318	0.1024	0.1339	0.1358	0.1365
SSP1-1.9	2060	0.2041	0.1516	0.2088	0.2106	0.2117
SSP1-1.9	2061	0.3581	0.2165	0.2189	0.4517	0.4569
SSP1-1.9	2062	0.9931	0.9862	0.9888	0.9962	1.0003
SSP1-1.9	2063	1.0008	0.9930	0.9979	1.0042	1.0080
SSP1-1.9	2064	0.9940	0.9871	0.9917	0.9965	1.0006
SSP1-1.9	2065	0.9629	0.4543	0.9915	0.9977	1.0022
SSP1-1.9	2066	0.9934	0.9894	0.9913	0.9948	0.9983
SSP1-1.9	2067	0.2125	0.1378	0.2179	0.2199	0.2210

Continue on the next page

**Scenario**	**Year**	**Average**	**Q5**	**Q25**	**Q75**	**Q95**
SSP1-1.9	2068	0.9464	0.4459	0.9867	0.9926	0.9987
SSP1-1.9	2069	0.3960	0.2182	0.4130	0.4561	0.4599
SSP1-1.9	2070	0.8795	0.3718	0.9849	0.9940	1.0625
SSP1-1.9	2071	0.9915	0.9869	0.9890	0.9938	0.9973
SSP1-1.9	2072	0.6690	0.2200	0.3748	0.9947	1.0672
SSP1-1.9	2073	0.7490	0.2218	0.4536	0.9963	1.0679
SSP1-1.9	2074	0.8823	0.3133	0.9898	0.9959	1.0666
SSP1-1.9	2075	0.5110	0.2650	0.5713	0.5759	0.5793
SSP1-1.9	2076	0.9126	0.3765	0.9914	0.9996	1.0662
SSP1-1.9	2077	0.8082	0.3735	0.4553	0.9964	1.0683
SSP1-1.9	2078	0.5318	0.4338	0.4357	0.5878	0.5924
SSP1-1.9	2079	1.0000	0.9928	0.9963	1.0038	1.0074
SSP1-1.9	2080	0.8200	0.4519	0.4544	0.9932	0.9971
SSP1-1.9	2081	0.2203	0.2174	0.2193	0.2215	0.2229
SSP1-1.9	2082	0.9222	0.3115	0.9248	0.9913	1.0582
SSP1-1.9	2083	0.1780	0.0898	0.1460	0.2167	0.2182
SSP1-1.9	2084	0.7265	0.3675	0.3715	1.0460	1.0557
SSP1-1.9	2085	0.9958	0.9900	0.9927	0.9983	1.0028
SSP1-1.9	2086	0.7928	0.3752	0.4579	1.0023	1.0094
SSP1-1.9	2087	0.9940	0.9886	0.9918	0.9962	0.9996
SSP1-1.9	2088	0.4152	0.1846	0.4517	0.4576	0.4604
SSP1-1.9	2089	0.8951	0.3731	0.9859	0.9940	1.0637
SSP1-1.9	2090	1.0012	0.9943	0.9981	1.0039	1.0094
SSP1-1.9	2091	0.9095	0.5174	0.9933	0.9980	1.0567
SSP1-1.9	2092	0.8675	0.3538	0.9904	0.9958	0.9991
SSP1-1.9	2093	0.7909	0.3514	0.4526	0.9943	0.9972
SSP1-1.9	2094	0.9966	0.9901	0.9939	0.9993	1.0033
SSP1-1.9	2095	0.9990	0.9915	0.9956	1.0021	1.0065
SSP1-1.9	2096	0.9935	0.9884	0.9912	0.9956	0.9992
SSP1-1.9	2097	0.8595	0.3747	0.8714	1.0121	1.0686
SSP1-1.9	2098	0.9978	0.9886	0.9946	1.0018	1.0060
SSP1-1.9	2099	0.9916	0.9868	0.9889	0.9940	0.9975
