# Supplementary material for: In hot water: Uncertainties in projecting marine heatwaves impacts on seagrass meadows
Source: PLoS One. 2024 Nov 27;19(11):e0298853. doi: 10.1371/journal.pone.0298853 (PMC11602073; doi:10.1371/journal.pone.0298853)
Supplement: S2 Table — Avg: denotes the average high shoot density ratio per decade. Q25: represents 25th percentile, marking the value below which 25% of the observations fall. Q95: stands for the 95th percentile indicating the value below which 95% of the observations are found. (PDF) [file pone.0298853.s010.pdf]

S2 Table. High Shoot Density Ratio Across Years for SSP1-2.6 Scenario: This table provides an analysis of the high shoot density states, measured annually within the SSP1-2.6 scenario. Avg: denotes the average high shoot density ratio per decade. **Q25:** represents 25th percentile, marking the value below which 25% of the observations fall. **Q95:** stands for the 95th percentile indicating the value below which 95% of the observations are found.

Scenario	Year	Average	Q5	Q25	Q75	Q95
SSP1-2.6	2030	0.8555	0.4484	0.9239	0.9928	0.9973
SSP1-2.6	2031	0.9941	0.9877	0.9911	0.9969	1.0008
SSP1-2.6	2032	0.2163	0.2145	0.2156	0.2170	0.2181
SSP1-2.6	2033	0.8970	0.3727	0.9840	0.9888	1.0547
SSP1-2.6	2034	0.9787	0.9866	0.9919	0.9982	1.0031
SSP1-2.6	2035	0.9514	0.4517	0.9886	0.9936	0.9982
SSP1-2.6	2036	0.9943	0.9891	0.9921	0.9965	0.9999
SSP1-2.6	2037	0.9986	0.9925	0.9961	1.0043	1.0088
SSP1-2.6	2038	0.2185	0.2183	0.2197	0.2213	0.2225
SSP1-2.6	2039	0.9871	0.9809	0.9846	0.9895	0.9929
SSP1-2.6	2040	0.2403	0.1372	0.2624	0.2650	0.2662
SSP1-2.6	2041	0.9604	0.9733	0.9799	0.9845	0.9881
SSP1-2.6	2042	0.3722	0.2179	0.2200	0.4518	0.4559
SSP1-2.6	2043	0.1913	0.1371	0.1389	0.2211	0.2226
SSP1-2.6	2044	0.9844	0.9739	0.9781	0.9891	0.9933
SSP1-2.6	2045	0.1819	0.1353	0.1365	0.2180	0.2192
SSP1-2.6	2046	0.9831	0.9724	0.9770	0.9886	0.9930
SSP1-2.6	2047	0.5828	0.2184	0.3725	1.0136	1.0696
SSP1-2.6	2048	0.3936	0.2169	0.2221	0.4570	0.4622
SSP1-2.6	2049	0.2182	0.2143	0.2167	0.2197	0.2210
SSP1-2.6	2050	0.4433	0.4376	0.4393	0.4430	0.4452
SSP1-2.6	2051	0.1348	0.1331	0.1341	0.1355	0.1365
SSP1-2.6	2052	0.2100	0.2079	0.2092	0.2108	0.2122
SSP1-2.6	2053	0.3549	0.2127	0.2143	0.4453	0.4490
SSP1-2.6	2054	0.4266	0.1674	0.4490	0.4583	0.4623
SSP1-2.6	2055	0.6697	0.1772	0.4547	0.9945	1.0017
SSP1-2.6	2056	0.3106	0.1146	0.2117	0.4520	0.4559
SSP1-2.6	2057	0.4108	0.2071	0.4283	0.4440	0.5878
SSP1-2.6	2058	0.9908	0.9819	0.9885	0.9941	0.9973
SSP1-2.6	2059	0.3806	0.1677	0.2193	0.4552	0.4568
SSP1-2.6	2060	0.7689	0.2691	0.3749	1.0503	1.0636
SSP1-2.6	2061	0.8581	0.3696	0.9865	0.9917	1.0592
SSP1-2.6	2062	0.2113	0.1364	0.2161	0.2178	0.2191
SSP1-2.6	2063	0.3985	0.1633	0.4338	0.4472	0.4498
SSP1-2.6	2064	0.2020	0.1053	0.2120	0.2159	0.2173
SSP1-2.6	2065	0.3257	0.1608	0.2135	0.4446	0.4488
SSP1-2.6	2066	0.3913	0.1882	0.3155	0.4507	0.4537
SSP1-2.6	2067	0.2086	0.1369	0.2161	0.2201	0.2211

Continue on the next page

Scenario	Year	Average	Q5	Q25	Q75	Q95
SSP1-2.6	2068	0.2631	0.1326	0.1755	0.3292	0.4451
SSP1-2.6	2069	0.2133	0.2075	0.2106	0.2161	0.2187
SSP1-2.6	2070	0.6873	0.2580	0.4251	0.9824	0.9863
SSP1-2.6	2071	0.3518	0.1671	0.2194	0.4534	0.4569
SSP1-2.6	2072	0.1797	0.0893	0.1470	0.2181	0.2202
SSP1-2.6	2073	0.4043	0.2654	0.4309	0.4416	0.4443
SSP1-2.6	2074	0.1853	0.1343	0.1356	0.2177	0.2188
SSP1-2.6	2075	0.3289	0.2021	0.2098	0.4437	0.4480
SSP1-2.6	2076	0.9810	0.9209	0.9833	0.9909	0.9938
SSP1-2.6	2077	0.3644	0.1480	0.2167	0.5733	0.5778
SSP1-2.6	2078	0.2588	0.2564	0.2603	0.2652	0.2665
SSP1-2.6	2079	0.7295	0.3069	0.3733	1.0529	1.0580
SSP1-2.6	2080	0.1763	0.1080	0.1384	0.2207	0.2227
SSP1-2.6	2081	0.9042	0.3671	0.9764	0.9872	1.0488
SSP1-2.6	2082	0.9280	0.4530	0.9894	0.9946	0.9975
SSP1-2.6	2083	0.4530	0.4494	0.4515	0.4545	0.4569
SSP1-2.6	2084	0.8184	0.3697	0.4528	0.9914	1.0506
SSP1-2.6	2085	0.9907	0.9871	0.9892	0.9925	0.9953
SSP1-2.6	2086	0.9539	0.4338	0.9904	0.9950	0.9978
SSP1-2.6	2087	0.8745	0.3727	0.9831	0.9903	0.9922
SSP1-2.6	2088	0.1803	0.1233	0.1366	0.2180	0.2199
SSP1-2.6	2089	0.2085	0.1308	0.1334	0.2586	0.2606
SSP1-2.6	2090	0.9780	0.9704	0.9736	0.9815	0.9857
SSP1-2.6	2091	0.9838	0.9258	0.9877	0.9931	0.9962
SSP1-2.6	2092	0.2100	0.1476	0.2163	0.2185	0.2201
SSP1-2.6	2093	0.4017	0.1617	0.4315	0.4459	0.4489
SSP1-2.6	2094	0.2049	0.1069	0.2155	0.2187	0.2201
SSP1-2.6	2095	0.2803	0.1334	0.1790	0.4457	0.4527
SSP1-2.6	2096	0.3850	0.1629	0.2157	0.4385	0.5886
SSP1-2.6	2097	0.1941	0.1252	0.1603	0.2200	0.2216
SSP1-2.6	2098	0.3383	0.1595	0.2137	0.4445	0.4477
SSP1-2.6	2099	0.2958	0.1349	0.1806	0.4494	0.4535
