# Supplementary material for: In hot water: Uncertainties in projecting marine heatwaves impacts on seagrass meadows
Source: PLoS One. 2024 Nov 27;19(11):e0298853. doi: 10.1371/journal.pone.0298853 (PMC11602073; doi:10.1371/journal.pone.0298853)
Supplement: S3 Table — Avg: denotes the average high shoot density ratio per decade. Q25: represents 25th percentile, marking the value below which 25% of the observations fall. Q95: stands for the 95th percentile indicating the value below which 95% of the observations are found. (PDF) [file pone.0298853.s011.pdf]

S3 Table. High Shoot Density Ratio Across Years for SSP3-7.0 Scenario: This table provides an analysis of the high shoot density states, measured annually within the SSP3-7.0 scenario. Avg: denotes the average high shoot density ratio per decade. **Q25:** represents 25th percentile, marking the value below which 25% of the observations fall. **Q95:** stands for the 95th percentile indicating the value below which 95% of the observations are found.

Scenario	Year	Average	Q5	Q25	Q75	Q95
SSP3-7.0	2030	0.7751	0.4618	0.4661	1.0212	1.0316
SSP3-7.0	2031	0.9838	0.6738	1.0132	1.0216	1.0483
SSP3-7.0	2032	0.9948	0.9895	0.9922	0.9973	1.0006
SSP3-7.0	2033	0.3764	0.2219	0.2241	0.4562	0.4600
SSP3-7.0	2034	0.9973	0.9880	0.9921	1.0017	1.0059
SSP3-7.0	2035	0.9927	0.9882	0.9905	0.9948	0.9981
SSP3-7.0	2036	0.9253	0.3806	1.0032	1.0124	1.0881
SSP3-7.0	2037	0.9233	0.4303	0.9918	0.9971	1.0008
SSP3-7.0	2038	1.0001	0.9916	0.9963	1.0031	1.0090
SSP3-7.0	2039	0.8769	0.3832	0.8712	1.0222	1.0975
SSP3-7.0	2040	0.9821	0.4619	1.0106	1.0188	1.0241
SSP3-7.0	2041	0.4286	0.2147	0.4510	0.4577	0.4624
SSP3-7.0	2042	0.9890	0.9810	0.9869	0.9918	0.9947
SSP3-7.0	2043	0.7333	0.4549	0.4572	0.9985	1.0033
SSP3-7.0	2044	0.9500	0.4515	0.9896	0.9956	1.0000
SSP3-7.0	2045	0.4176	0.2200	0.4529	0.4601	0.4644
SSP3-7.0	2046	0.9868	0.9240	0.9895	0.9953	0.9997
SSP3-7.0	2047	0.7919	0.3727	0.3945	1.0057	1.0759
SSP3-7.0	2048	0.9899	0.9849	0.9874	0.9922	0.9967
SSP3-7.0	2049	0.9807	0.9919	0.9986	1.0066	1.0127
SSP3-7.0	2050	0.2128	0.1694	0.2184	0.2202	0.2215
SSP3-7.0	2051	0.2001	0.1243	0.2112	0.2140	0.2154
SSP3-7.0	2052	0.6078	0.2118	0.3690	1.0512	1.0576
SSP3-7.0	2053	0.3413	0.1357	0.1826	0.4539	0.4586
SSP3-7.0	2054	0.4117	0.2144	0.4336	0.4454	0.5881
SSP3-7.0	2055	0.8084	0.4484	0.4539	0.9933	0.9965
SSP3-7.0	2056	0.1991	0.1034	0.1354	0.2624	0.2641
SSP3-7.0	2057	0.3624	0.2031	0.2412	0.4378	0.4429
SSP3-7.0	2058	0.4106	0.1649	0.4415	0.4509	0.4542
SSP3-7.0	2059	0.5097	0.2664	0.4326	0.5783	0.5883
SSP3-7.0	2060	0.1971	0.0996	0.2060	0.2155	0.2173
SSP3-7.0	2061	0.1770	0.1123	0.1314	0.2102	0.2116
SSP3-7.0	2062	0.5367	0.1546	0.3199	0.9768	1.0428
SSP3-7.0	2063	0.3100	0.0870	0.2052	0.4383	0.5869
SSP3-7.0	2064	0.1703	0.0861	0.1322	0.2133	0.2164
SSP3-7.0	2065	0.1938	0.1067	0.2001	0.2123	0.2136
SSP3-7.0	2066	0.5929	0.1614	0.3577	0.9839	1.0498
SSP3-7.0	2067	0.2045	0.1051	0.2128	0.2161	0.2175

Continue on the next page

**Scenario**	**Year**	**Average**	**Q5**	**Q25**	**Q75**	**Q95**
SSP3-7.0	2068	0.1922	0.1199	0.2061	0.2092	0.2108
SSP3-7.0	2069	0.2005	0.1582	0.2091	0.2118	0.2133
SSP3-7.0	2070	0.1415	0.0734	0.0984	0.2001	0.2095
SSP3-7.0	2071	0.1108	0.0762	0.1003	0.1288	0.1309
SSP3-7.0	2072	0.1162	0.0759	0.1192	0.1262	0.1273
SSP3-7.0	2073	0.3509	0.1854	0.2629	0.4209	0.4232
SSP3-7.0	2074	0.1782	0.0970	0.1397	0.2115	0.2124
SSP3-7.0	2075	0.1313	0.0732	0.0965	0.1623	0.1652
SSP3-7.0	2076	0.0992	0.0976	0.0984	0.1001	0.1011
SSP3-7.0	2077	0.1637	0.1242	0.1253	0.1988	0.2006
SSP3-7.0	2078	0.0990	0.0972	0.0983	0.1009	0.1016
SSP3-7.0	2079	0.0906	0.0752	0.0759	0.0981	0.0991
SSP3-7.0	2080	0.1437	0.0757	0.1105	0.1970	0.1987
SSP3-7.0	2081	0.1233	0.0992	0.1250	0.1286	0.1295
SSP3-7.0	2082	0.1161	0.0949	0.0972	0.1264	0.1271
SSP3-7.0	2083	0.1074	0.0747	0.0761	0.1251	0.1267
SSP3-7.0	2084	0.0893	0.0748	0.0757	0.0978	0.0984
SSP3-7.0	2085	0.0892	0.0764	0.0769	0.0994	0.1004
SSP3-7.0	2086	0.1082	0.0756	0.0990	0.1246	0.1259
SSP3-7.0	2087	0.1699	0.0853	0.1132	0.1992	0.2009
SSP3-7.0	2088	0.0922	0.0743	0.0758	0.1002	0.1010
SSP3-7.0	2089	0.1130	0.0717	0.0729	0.1390	0.1403
SSP3-7.0	2090	0.1414	0.0725	0.0874	0.1952	0.2040
SSP3-7.0	2091	0.1032	0.0725	0.0749	0.1236	0.1255
SSP3-7.0	2092	0.1120	0.0705	0.0827	0.1517	0.1553
SSP3-7.0	2093	0.0745	0.0620	0.0747	0.0759	0.0778
SSP3-7.0	2094	0.0744	0.0735	0.0741	0.0748	0.0754
SSP3-7.0	2095	0.0738	0.0607	0.0744	0.0754	0.0772
SSP3-7.0	2096	0.0747	0.0738	0.0747	0.0755	0.0762
SSP3-7.0	2097	0.0749	0.0738	0.0744	0.0754	0.0762
SSP3-7.0	2098	0.0761	0.0751	0.0757	0.0765	0.0772
SSP3-7.0	2099	0.0761	0.0751	0.0757	0.0765	0.0773
