# Supplementary material for: In hot water: Uncertainties in projecting marine heatwaves impacts on seagrass meadows
Source: PLoS One. 2024 Nov 27;19(11):e0298853. doi: 10.1371/journal.pone.0298853 (PMC11602073; doi:10.1371/journal.pone.0298853)
Supplement: S4 Table — Avg: denotes the average high shoot density ratio per decade. Q25: represents 25th percentile, marking the value below which 25% of the observations fall. Q95: stands for the 95th percentile indicating the value below which 95% of the observations are found. (PDF) [file pone.0298853.s012.pdf]

S4 Table. High Shoot Density Ratio Across Years for SSP5-8.5 Scenario: This table provides an analysis of the high shoot density states, measured annually within the SSP5-8.5 scenario. Avg: denotes the average high shoot density ratio per decade. **Q25:** represents 25th percentile, marking the value below which 25% of the observations fall. **Q95:** stands for the 95th percentile indicating the value below which 95% of the observations are found.

Scenario	Year	Average	Q5	Q25	Q75	Q95
SSP5-8.5	2030	0.3759	0.1365	0.2972	0.4540	0.4568
SSP5-8.5	2031	0.7574	0.3870	0.4529	0.9986	1.0272
SSP5-8.5	2032	0.3881	0.2214	0.2238	0.4603	0.4640
SSP5-8.5	2033	0.7443	0.1707	0.4516	0.9964	1.0678
SSP5-8.5	2034	0.2125	0.1495	0.2170	0.2189	0.2204
SSP5-8.5	2035	0.9855	0.9787	0.9831	0.9881	0.9915
SSP5-8.5	2036	0.9866	0.9905	0.9951	1.0005	1.0045
SSP5-8.5	2037	0.9354	0.4522	0.9910	0.9972	1.0030
SSP5-8.5	2038	0.9900	0.9859	0.9878	0.9914	0.9948
SSP5-8.5	2039	0.3522	0.2169	0.2183	0.4533	0.4558
SSP5-8.5	2040	0.3401	0.2112	0.2147	0.4494	0.4523
SSP5-8.5	2041	0.4238	0.2158	0.4444	0.4526	0.4551
SSP5-8.5	2042	0.4376	0.2202	0.4532	0.4589	0.4637
SSP5-8.5	2043	0.3127	0.1360	0.1822	0.4526	0.4574
SSP5-8.5	2044	0.2031	0.1344	0.2113	0.2185	0.2205
SSP5-8.5	2045	0.3897	0.2083	0.4293	0.4424	0.4451
SSP5-8.5	2046	0.1974	0.1334	0.1358	0.2632	0.2644
SSP5-8.5	2047	0.2095	0.2058	0.2078	0.2115	0.2130
SSP5-8.5	2048	0.1329	0.1316	0.1324	0.1335	0.1342
SSP5-8.5	2049	0.3677	0.2076	0.2109	0.4307	0.4332
SSP5-8.5	2050	0.1327	0.1307	0.1315	0.1337	0.1344
SSP5-8.5	2051	0.1799	0.1554	0.1566	0.2041	0.2055
SSP5-8.5	2052	0.1073	0.0806	0.0912	0.1312	0.1330
SSP5-8.5	2053	0.1179	0.0964	0.0974	0.1279	0.1288
SSP5-8.5	2054	0.1543	0.1011	0.1192	0.2036	0.2053
SSP5-8.5	2055	0.1845	0.0991	0.1502	0.2094	0.2119
SSP5-8.5	2056	0.1585	0.0867	0.1007	0.2083	0.2108
SSP5-8.5	2057	0.1629	0.0857	0.1057	0.2077	0.2103
SSP5-8.5	2058	0.0915	0.0760	0.0769	0.1018	0.1027
SSP5-8.5	2059	0.1175	0.0770	0.0997	0.1430	0.1451
SSP5-8.5	2060	0.1472	0.0736	0.1192	0.1972	0.2031
SSP5-8.5	2061	0.1207	0.0986	0.1234	0.1290	0.1305
SSP5-8.5	2062	0.2925	0.1515	0.1562	0.4273	0.4311
SSP5-8.5	2063	0.1166	0.0755	0.1001	0.1314	0.1326
SSP5-8.5	2064	0.1743	0.0850	0.1437	0.2015	0.2031
SSP5-8.5	2065	0.0970	0.0695	0.0985	0.1014	0.1042
SSP5-8.5	2066	0.0909	0.0765	0.0772	0.0999	0.1010
SSP5-8.5	2067	0.1075	0.0755	0.0774	0.1248	0.1261

Continue on the next page

Scenario	Year	Average	Q5	Q25	Q75	Q95
SSP5-8.5	2068	0.0905	0.0745	0.0757	0.0971	0.0980
SSP5-8.5	2069	0.0888	0.0764	0.0770	0.0998	0.1007
SSP5-8.5	2070	0.1091	0.0761	0.0982	0.1242	0.1254
SSP5-8.5	2071	0.0955	0.0762	0.0969	0.0983	0.0999
SSP5-8.5	2072	0.0915	0.0758	0.0766	0.0992	0.1006
SSP5-8.5	2073	0.0877	0.0726	0.0738	0.0954	0.0962
SSP5-8.5	2074	0.0854	0.0624	0.0758	0.0985	0.0997
SSP5-8.5	2075	0.1003	0.0754	0.0771	0.1237	0.1253
SSP5-8.5	2076	0.0827	0.0598	0.0732	0.0961	0.0983
SSP5-8.5	2077	0.0752	0.0628	0.0756	0.0768	0.0793
SSP5-8.5	2078	0.0734	0.0608	0.0745	0.0755	0.0777
SSP5-8.5	2079	0.0722	0.0602	0.0737	0.0756	0.0775
SSP5-8.5	2080	0.0720	0.0456	0.0726	0.0742	0.0872
SSP5-8.5	2081	0.0705	0.0368	0.0729	0.0742	0.0866
SSP5-8.5	2082	0.0685	0.0500	0.0596	0.0750	0.0762
SSP5-8.5	2083	0.0692	0.0595	0.0607	0.0760	0.0769
SSP5-8.5	2084	0.0730	0.0607	0.0733	0.0746	0.0770
SSP5-8.5	2085	0.0599	0.0589	0.0598	0.0609	0.0617
SSP5-8.5	2086	0.0684	0.0578	0.0593	0.0749	0.0757
SSP5-8.5	2087	0.0696	0.0579	0.0713	0.0730	0.0750
SSP5-8.5	2088	0.0723	0.0601	0.0735	0.0749	0.0769
SSP5-8.5	2089	0.0731	0.0583	0.0732	0.0744	0.0861
SSP5-8.5	2090	0.0719	0.0500	0.0733	0.0744	0.0759
SSP5-8.5	2091	0.0726	0.0602	0.0737	0.0755	0.0776
SSP5-8.5	2092	0.0709	0.0449	0.0731	0.0751	0.0874
SSP5-8.5	2093	0.0590	0.0477	0.0601	0.0616	0.0626
SSP5-8.5	2094	0.0684	0.0406	0.0680	0.0733	0.0865
SSP5-8.5	2095	0.0668	0.0340	0.0697	0.0725	0.0860
SSP5-8.5	2096	0.0663	0.0340	0.0585	0.0735	0.0866
SSP5-8.5	2097	0.0593	0.0340	0.0556	0.0615	0.0839
SSP5-8.5	2098	0.0580	0.0323	0.0533	0.0603	0.0836
SSP5-8.5	2099	0.0560	0.0449	0.0557	0.0586	0.0589
