# Supplementary material for: In hot water: Uncertainties in projecting marine heatwaves impacts on seagrass meadows
Source: PLoS One. 2024 Nov 27;19(11):e0298853. doi: 10.1371/journal.pone.0298853 (PMC11602073; doi:10.1371/journal.pone.0298853)
Supplement: S5 Table — Avg: denotes the average moderate shoot density ratio per decade. Q25: represents 25th percentile, marking the value below which 25% of the observations fall. Q95: stands for the 95th percentile indicating the value below which 95% of the observations are found. (PDF) [file pone.0298853.s013.pdf]

S5 Table. Moderate Shoot Density Ratio Across Years for SSP1-1.9 Scenario: This table provides an analysis of the moderate shoot density states, measured annually within the SSP1-1.9 scenario.Avg: denotes the average moderate shoot density ratio per decade. **Q25:** represents 25th percentile, marking the value below which 25% of the observations fall. **Q95:** stands for the 95th percentile indicating the value below which 95% of the observations are found.

**Scenario**	**Year**	**Average**	**Q5**	**Q25**	**Q75**	**Q95**
SSP1-1.9	2030	1.0221	0.7470	1.0505	1.0560	1.1470
SSP1-1.9	2031	1.0219	0.9674	0.9978	1.0298	1.1572
SSP1-1.9	2032	1.0130	0.9622	1.0025	1.0061	1.0955
SSP1-1.9	2033	1.0075	1.0026	1.0049	1.0080	1.0100
SSP1-1.9	2034	1.0057	1.0018	1.0039	1.0073	1.0097
SSP1-1.9	2035	0.8781	0.6975	0.7000	1.0273	1.0302
SSP1-1.9	2036	1.0085	1.0032	1.0056	1.0111	1.0135
SSP1-1.9	2037	0.9871	0.8810	0.9126	1.0208	1.0869
SSP1-1.9	2038	0.8896	0.7009	0.7026	1.0311	1.0344
SSP1-1.9	2039	1.0170	0.9564	0.9647	1.0901	1.1074
SSP1-1.9	2040	1.0062	1.0023	1.0047	1.0078	1.0099
SSP1-1.9	2041	0.7885	0.7849	0.7871	0.7898	0.7926
SSP1-1.9	2042	0.9070	0.6567	0.7319	1.0297	1.0729
SSP1-1.9	2043	0.9772	0.6585	0.8951	1.0543	1.2111
SSP1-1.9	2044	0.9447	0.6523	0.6926	1.1591	1.2206
SSP1-1.9	2045	0.8908	0.6944	0.7049	1.0325	1.0401
SSP1-1.9	2046	1.0167	0.9609	0.9895	1.0319	1.1116
SSP1-1.9	2047	0.9317	0.6852	0.9429	0.9475	1.0693
SSP1-1.9	2048	0.9425	0.9373	0.9451	0.9495	0.9564
SSP1-1.9	2049	1.0169	0.9607	0.9807	1.0614	1.1106
SSP1-1.9	2050	0.9477	0.9438	0.9460	0.9493	0.9518
SSP1-1.9	2051	0.9851	0.6752	0.8778	1.0914	1.1736
SSP1-1.9	2052	1.0078	1.0017	1.0040	1.0072	1.0448
SSP1-1.9	2053	1.0222	0.9637	0.9662	1.0962	1.1007
SSP1-1.9	2054	0.8854	0.6980	0.7032	1.0295	1.0356
SSP1-1.9	2055	1.0042	1.0007	1.0037	1.0101	1.0127
SSP1-1.9	2056	1.0063	1.0022	1.0045	1.0080	1.0102
SSP1-1.9	2057	1.0056	1.0022	1.0044	1.0070	1.0090
SSP1-1.9	2058	0.8819	0.7021	0.7049	1.0331	1.0370
SSP1-1.9	2059	0.7259	0.7049	0.7235	0.7310	0.7333
SSP1-1.9	2060	0.7690	0.6495	0.7746	0.7779	0.8304
SSP1-1.9	2061	0.8779	0.6913	0.6962	1.0269	1.0325
SSP1-1.9	2062	1.0088	1.0034	1.0060	1.0120	1.0145
SSP1-1.9	2063	1.0065	1.0024	1.0047	1.0084	1.0109
SSP1-1.9	2064	1.0058	1.0017	1.0042	1.0073	1.0097
SSP1-1.9	2065	1.0013	0.9001	1.0030	1.0067	1.0484
SSP1-1.9	2066	1.0059	1.0025	1.0047	1.0074	1.0092
SSP1-1.9	2067	0.7829	0.6368	0.7861	0.7895	0.8750

Continue on the next page

Scenario	Year	Average	Q5	Q25	Q75	Q95
SSP1-1.9	2068	1.0058	0.9789	1.0057	1.0095	1.0558
SSP1-1.9	2069	0.9071	0.7000	0.8917	0.9691	1.0358
SSP1-1.9	2070	1.0055	0.8802	1.0049	1.0116	1.0991
SSP1-1.9	2071	1.0071	1.0035	1.0056	1.0086	1.0110
SSP1-1.9	2072	0.9741	0.6764	0.8989	1.0536	1.1766
SSP1-1.9	2073	0.9832	0.6772	0.9011	1.0495	1.1743
SSP1-1.9	2074	1.0122	0.8814	1.0072	1.0114	1.1357
SSP1-1.9	2075	0.9989	0.7521	1.0392	1.0447	1.1033
SSP1-1.9	2076	1.0130	0.9666	1.0024	1.0082	1.1081
SSP1-1.9	2077	0.9942	0.8767	0.9684	1.0486	1.0988
SSP1-1.9	2078	1.0484	1.0170	1.0207	1.0806	1.0855
SSP1-1.9	2079	1.0041	1.0002	1.0022	1.0059	1.0083
SSP1-1.9	2080	0.9860	0.9007	0.9025	1.0470	1.0504
SSP1-1.9	2081	0.7907	0.7850	0.7880	0.7931	0.7973
SSP1-1.9	2082	1.0235	0.9648	1.0060	1.0438	1.1221
SSP1-1.9	2083	0.7527	0.5945	0.6386	0.8558	0.8915
SSP1-1.9	2084	1.0165	0.9389	0.9633	1.0849	1.1263
SSP1-1.9	2085	1.0065	1.0024	1.0048	1.0082	1.0104
SSP1-1.9	2086	0.9824	0.8984	0.9017	1.0476	1.0585
SSP1-1.9	2087	1.0093	1.0054	1.0077	1.0108	1.0133
SSP1-1.9	2088	0.9162	0.6544	0.9421	0.9479	1.0734
SSP1-1.9	2089	1.0041	0.8758	1.0016	1.0077	1.0957
SSP1-1.9	2090	1.0044	0.9999	1.0027	1.0060	1.0088
SSP1-1.9	2091	1.0131	0.9756	1.0032	1.0074	1.0719
SSP1-1.9	2092	0.9920	0.8810	1.0052	1.0101	1.0891
SSP1-1.9	2093	0.9818	0.8816	0.9098	1.0133	1.0906
SSP1-1.9	2094	1.0046	1.0002	1.0028	1.0062	1.0086
SSP1-1.9	2095	1.0046	1.0005	1.0028	1.0063	1.0086
SSP1-1.9	2096	1.0055	1.0017	1.0039	1.0070	1.0092
SSP1-1.9	2097	1.0180	0.9636	0.9961	1.0212	1.0987
SSP1-1.9	2098	1.0033	0.9985	1.0012	1.0052	1.0083
SSP1-1.9	2099	1.0066	1.0031	1.0050	1.0082	1.0102
