# Supplementary material for: In hot water: Uncertainties in projecting marine heatwaves impacts on seagrass meadows
Source: PLoS One. 2024 Nov 27;19(11):e0298853. doi: 10.1371/journal.pone.0298853 (PMC11602073; doi:10.1371/journal.pone.0298853)
Supplement: S6 Table — Avg: denotes the average moderate shoot density ratio per decade. Q25: represents 25th percentile, marking the value below which 25% of the observations fall. Q95: stands for the 95th percentile indicating the value below which 95% of the observations are found. (PDF) [file pone.0298853.s014.pdf]

S6 Table. Moderate Shoot Density Ratio Across Years for SSP1-2.6 Scenario: This table provides an analysis of the moderate shoot density states, measured annually within the SSP1-2.6 scenario. Avg: denotes the average moderate shoot density ratio per decade. **Q25:** represents 25th percentile, marking the value below which 25% of the observations fall. **Q95:** stands for the 95th percentile indicating the value below which 95% of the observations are found.

Scenario	Year	Average	Q5	Q25	Q75	Q95
SSP1-2.6	2030	0.9930	0.9017	1.0052	1.0091	1.0523
SSP1-2.6	2031	1.0061	1.0018	1.0044	1.0079	1.0100
SSP1-2.6	2032	0.7913	0.7880	0.7898	0.7927	0.7949
SSP1-2.6	2033	1.0134	0.9615	1.0063	1.0105	1.0973
SSP1-2.6	2034	1.0097	1.0030	1.0061	1.0101	1.0129
SSP1-2.6	2035	1.0023	0.9025	1.0054	1.0088	1.0506
SSP1-2.6	2036	1.0070	1.0030	1.0053	1.0086	1.0110
SSP1-2.6	2037	1.0064	1.0006	1.0033	1.0075	1.0102
SSP1-2.6	2038	0.7841	0.7823	0.7849	0.7887	0.7919
SSP1-2.6	2039	1.0098	1.0059	1.0084	1.0113	1.0134
SSP1-2.6	2040	0.7937	0.6728	0.8085	0.8120	0.8620
SSP1-2.6	2041	1.0082	1.0063	1.0088	1.0122	1.0164
SSP1-2.6	2042	0.9623	0.6807	0.6845	1.1605	1.1666
SSP1-2.6	2043	0.7632	0.6303	0.6355	0.8735	0.8781
SSP1-2.6	2044	1.0110	1.0053	1.0077	1.0149	1.0178
SSP1-2.6	2045	0.7616	0.6382	0.6402	0.8754	0.8789
SSP1-2.6	2046	1.0076	1.0011	1.0041	1.0116	1.0141
SSP1-2.6	2047	0.9571	0.6762	0.8930	1.0663	1.1794
SSP1-2.6	2048	0.9062	0.6959	0.7073	1.0286	1.0388
SSP1-2.6	2049	0.7860	0.7759	0.7802	0.7899	0.7939
SSP1-2.6	2050	1.0408	1.0363	1.0389	1.0431	1.0458
SSP1-2.6	2051	0.7306	0.7265	0.7289	0.7323	0.7347
SSP1-2.6	2052	0.7750	0.7711	0.7734	0.7766	0.7789
SSP1-2.6	2053	0.8840	0.6967	0.6995	1.0320	1.0355
SSP1-2.6	2054	0.9331	0.6925	0.9406	0.9509	1.0505
SSP1-2.6	2055	0.9655	0.6921	0.9023	1.0487	1.1634
SSP1-2.6	2056	0.8636	0.5963	0.7065	1.0456	1.0998
SSP1-2.6	2057	0.9875	0.6517	0.9838	1.0605	1.1965
SSP1-2.6	2058	1.0075	1.0030	1.0054	1.0090	1.0140
SSP1-2.6	2059	0.9043	0.7010	0.7033	1.0322	1.0495
SSP1-2.6	2060	1.0121	0.7456	0.9704	1.0612	1.1408
SSP1-2.6	2061	0.9998	0.8799	1.0062	1.0107	1.1090
SSP1-2.6	2062	0.7856	0.6392	0.7883	0.7918	0.8773
SSP1-2.6	2063	0.9216	0.6949	0.9442	0.9673	1.0511
SSP1-2.6	2064	0.7808	0.6385	0.7828	0.7958	0.8572
SSP1-2.6	2065	0.8716	0.6911	0.6984	1.0326	1.0522
SSP1-2.6	2066	0.9110	0.6616	0.7816	1.0086	1.0856
SSP1-2.6	2067	0.7775	0.6332	0.7785	0.7900	0.8754
SSP1-2.6	2068	0.8214	0.5572	0.7209	0.9126	1.1268

Continue on the next page

**Scenario**	**Year**	**Average**	**Q5**	**Q25**	**Q75**	**Q95**
SSP1-2.6	2069	0.7825	0.7662	0.7754	0.7901	0.7993
SSP1-2.6	2070	0.9673	0.6904	0.8975	1.0554	1.1516
SSP1-2.6	2071	0.8849	0.6965	0.7017	1.0303	1.0498
SSP1-2.6	2072	0.7629	0.5903	0.6717	0.8489	0.8931
SSP1-2.6	2073	0.9880	0.7542	1.0102	1.0421	1.1008
SSP1-2.6	2074	0.7676	0.6392	0.6423	0.8793	0.8830
SSP1-2.6	2075	0.8666	0.6797	0.6916	1.0361	1.0488
SSP1-2.6	2076	1.0171	1.0079	1.0108	1.0168	1.0520
SSP1-2.6	2077	0.8947	0.5394	0.6884	1.1434	1.2088
SSP1-2.6	2078	0.8076	0.8029	0.8064	0.8138	0.8183
SSP1-2.6	2079	1.0101	0.8764	0.9569	1.0836	1.1214
SSP1-2.6	2080	0.7470	0.5354	0.6375	0.8744	0.9152
SSP1-2.6	2081	1.0060	0.8820	1.0063	1.0143	1.0895
SSP1-2.6	2082	0.9989	0.9009	1.0044	1.0079	1.0518
SSP1-2.6	2083	0.9497	0.9458	0.9481	0.9512	0.9535
SSP1-2.6	2084	0.9913	0.8830	0.9918	1.0480	1.0664
SSP1-2.6	2085	1.0070	1.0035	1.0055	1.0083	1.0106
SSP1-2.6	2086	1.0046	0.8936	1.0078	1.0111	1.0511
SSP1-2.6	2087	1.0020	0.8941	1.0087	1.0182	1.0823
SSP1-2.6	2088	0.7540	0.5908	0.6397	0.8747	0.8912
SSP1-2.6	2089	0.7785	0.6663	0.6752	0.8647	0.8711
SSP1-2.6	2090	1.0122	1.0065	1.0095	1.0155	1.0178
SSP1-2.6	2091	1.0150	1.0071	1.0096	1.0133	1.0477
SSP1-2.6	2092	0.7784	0.6331	0.7858	0.7904	0.8544
SSP1-2.6	2093	0.9289	0.6985	0.9476	0.9715	1.0518
SSP1-2.6	2094	0.7775	0.6336	0.7795	0.7909	0.8540
SSP1-2.6	2095	0.8297	0.5536	0.6612	1.0709	1.1307
SSP1-2.6	2096	0.9458	0.6653	0.6791	1.1488	1.1993
SSP1-2.6	2097	0.7646	0.5888	0.6784	0.8423	0.8892
SSP1-2.6	2098	0.8786	0.6880	0.7009	1.0333	1.0562
SSP1-2.6	2099	0.8387	0.5514	0.6584	1.0705	1.1247
