# Supplementary material for: In hot water: Uncertainties in projecting marine heatwaves impacts on seagrass meadows
Source: PLoS One. 2024 Nov 27;19(11):e0298853. doi: 10.1371/journal.pone.0298853 (PMC11602073; doi:10.1371/journal.pone.0298853)
Supplement: S7 Table — Avg: denotes the average moderate shoot density ratio per decade. Q25: represents 25th percentile, marking the value below which 25% of the observations fall. Q95: stands for the 95th percentile indicating the value below which 95% of the observations are found. (PDF) [file pone.0298853.s015.pdf]

S7 Table. Moderate Shoot Density Ratio Across Years for SSP3-7.0 Scenario: This table provides an analysis of the moderate shoot density states, measured annually within the SSP3-7.0 scenario. Avg: denotes the average moderate shoot density ratio per decade. **Q25:** represents 25th percentile, marking the value below which 25% of the observations fall. **Q95:** stands for the 95th percentile indicating the value below which 95% of the observations are found.

Scenario	Year	Average	Q5	Q25	Q75	Q95
SSP3-7.0	2030	0.9799	0.8961	0.8999	1.0522	1.0590
SSP3-7.0	2031	1.0069	0.9891	0.9998	1.0040	1.0311
SSP3-7.0	2032	1.0065	1.0026	1.0048	1.0082	1.0103
SSP3-7.0	2033	0.9621	0.6773	0.6811	1.1565	1.1636
SSP3-7.0	2034	1.0061	1.0005	1.0032	1.0097	1.0124
SSP3-7.0	2035	1.0055	1.0023	1.0042	1.0068	1.0087
SSP3-7.0	2036	1.0114	0.9610	1.0003	1.0055	1.1004
SSP3-7.0	2037	0.9967	0.8883	1.0037	1.0075	1.0516
SSP3-7.0	2038	1.0048	1.0001	1.0030	1.0066	1.0098
SSP3-7.0	2039	1.0132	0.9576	0.9948	1.0155	1.1011
SSP3-7.0	2040	1.0013	0.8995	1.0026	1.0065	1.0544
SSP3-7.0	2041	0.9364	0.7074	0.9508	0.9565	1.0434
SSP3-7.0	2042	1.0070	1.0029	1.0051	1.0085	1.0129
SSP3-7.0	2043	0.9762	0.8997	0.9021	1.0490	1.0528
SSP3-7.0	2044	1.0043	0.9043	1.0072	1.0109	1.0548
SSP3-7.0	2045	0.9199	0.7002	0.9436	0.9504	1.0347
SSP3-7.0	2046	1.0097	1.0016	1.0045	1.0082	1.0460
SSP3-7.0	2047	1.0064	0.8757	0.9893	1.0562	1.1586
SSP3-7.0	2048	1.0075	1.0034	1.0060	1.0091	1.0109
SSP3-7.0	2049	1.0097	1.0017	1.0055	1.0096	1.0134
SSP3-7.0	2050	0.7883	0.7833	0.7855	0.7893	0.8052
SSP3-7.0	2051	0.7725	0.5969	0.7836	0.7893	0.8832
SSP3-7.0	2052	0.9927	0.6767	0.9555	1.0877	1.1758
SSP3-7.0	2053	0.8709	0.5537	0.6601	1.0727	1.1254
SSP3-7.0	2054	0.9980	0.6721	1.0214	1.0580	1.1820
SSP3-7.0	2055	0.9872	0.9023	0.9043	1.0495	1.0557
SSP3-7.0	2056	0.7726	0.6749	0.6769	0.8645	0.8668
SSP3-7.0	2057	0.9005	0.6918	0.7623	1.0162	1.0520
SSP3-7.0	2058	0.9301	0.7030	0.9443	0.9544	1.0524
SSP3-7.0	2059	1.0259	0.7519	1.0208	1.0775	1.1379
SSP3-7.0	2060	0.7832	0.6411	0.7911	0.8084	0.8833
SSP3-7.0	2061	0.7590	0.5920	0.6428	0.8703	0.8817
SSP3-7.0	2062	0.9511	0.6738	0.8742	1.0827	1.2082
SSP3-7.0	2063	0.8986	0.4967	0.6799	1.1713	1.2940
SSP3-7.0	2064	0.7570	0.5877	0.6429	0.8748	0.9021
SSP3-7.0	2065	0.7720	0.6438	0.7727	0.8006	0.8461
SSP3-7.0	2066	0.9624	0.6783	0.9008	1.0699	1.2087
SSP3-7.0	2067	0.7853	0.6375	0.7857	0.7956	0.8588

Continue on the next page

Scenario	Year	Average	Q5	Q25	Q75	Q95
SSP3-7.0	2068	0.7740	0.6005	0.7840	0.7935	0.8910
SSP3-7.0	2069	0.7862	0.7709	0.7833	0.7883	0.8031
SSP3-7.0	2070	0.7415	0.5482	0.6552	0.8581	0.9098
SSP3-7.0	2071	0.6841	0.5973	0.6229	0.7546	0.7616
SSP3-7.0	2072	0.7097	0.6034	0.7199	0.7271	0.7549
SSP3-7.0	2073	0.9429	0.6485	0.7973	1.0568	1.1581
SSP3-7.0	2074	0.7774	0.6422	0.6791	0.8450	0.8843
SSP3-7.0	2075	0.7523	0.5569	0.6536	0.8519	0.8883
SSP3-7.0	2076	0.6539	0.6489	0.6512	0.6570	0.6599
SSP3-7.0	2077	0.7545	0.6424	0.6450	0.8590	0.8624
SSP3-7.0	2078	0.7012	0.6952	0.6975	0.7095	0.7121
SSP3-7.0	2079	0.6459	0.6178	0.6196	0.6597	0.6623
SSP3-7.0	2080	0.7286	0.5546	0.6090	0.8595	0.9059
SSP3-7.0	2081	0.7164	0.6238	0.7184	0.7340	0.7614
SSP3-7.0	2082	0.7153	0.6905	0.6990	0.7264	0.7316
SSP3-7.0	2083	0.6948	0.6014	0.6051	0.7424	0.7566
SSP3-7.0	2084	0.6713	0.6026	0.6049	0.7162	0.7189
SSP3-7.0	2085	0.6398	0.6169	0.6188	0.6576	0.6601
SSP3-7.0	2086	0.6877	0.6034	0.6303	0.7480	0.7516
SSP3-7.0	2087	0.7681	0.6079	0.7021	0.8143	0.8835
SSP3-7.0	2088	0.6816	0.5971	0.6013	0.7248	0.7279
SSP3-7.0	2089	0.6941	0.5767	0.5814	0.7616	0.7942
SSP3-7.0	2090	0.7340	0.5501	0.6133	0.8503	0.9148
SSP3-7.0	2091	0.6909	0.5982	0.6033	0.7538	0.7677
SSP3-7.0	2092	0.7194	0.5581	0.6158	0.8393	0.8766
SSP3-7.0	2093	0.6170	0.5673	0.6163	0.6214	0.6421
SSP3-7.0	2094	0.6213	0.6183	0.6202	0.6224	0.6247
SSP3-7.0	2095	0.6181	0.5686	0.6191	0.6223	0.6437
SSP3-7.0	2096	0.6201	0.6172	0.6196	0.6221	0.6251
SSP3-7.0	2097	0.6211	0.6172	0.6195	0.6226	0.6246
SSP3-7.0	2098	0.6206	0.6172	0.6194	0.6217	0.6237
SSP3-7.0	2099	0.6211	0.6177	0.6199	0.6223	0.6243
