# Supplementary material for: In hot water: Uncertainties in projecting marine heatwaves impacts on seagrass meadows
Source: PLoS One. 2024 Nov 27;19(11):e0298853. doi: 10.1371/journal.pone.0298853 (PMC11602073; doi:10.1371/journal.pone.0298853)
Supplement: S8 Table — Avg: denotes the average moderate shoot density ratio per decade. Q25: represents 25th percentile, marking the value below which 25% of the observations fall. Q95: stands for the 95th percentile indicating the value below which 95% of the observations are found. (PDF) [file pone.0298853.s016.pdf]

S8 Table. Moderate Shoot Density Ratio Across Years for SSP5-8.5 Scenario: This table provides an analysis of the moderate shoot density states, measured annually within the SSP5-8.5 scenario. Avg: denotes the average moderate shoot density ratio per decade. **Q25:** represents 25th percentile, marking the value below which 25% of the observations fall. **Q95:** stands for the 95th percentile indicating the value below which 95% of the observations are found.

Scenario	Year	Average	Q5	Q25	Q75	Q95
SSP5-8.5	2030	0.9006	0.6546	0.7328	1.0305	1.0807
SSP5-8.5	2031	0.9825	0.8890	0.9012	1.0482	1.1184
SSP5-8.5	2032	0.8973	0.6990	0.7028	1.0278	1.0330
SSP5-8.5	2033	0.9878	0.6804	0.9029	1.0507	1.1924
SSP5-8.5	2034	0.7821	0.6353	0.7873	0.7918	0.8555
SSP5-8.5	2035	1.0079	1.0037	1.0063	1.0096	1.0128
SSP5-8.5	2036	1.0042	1.0016	1.0040	1.0073	1.0095
SSP5-8.5	2037	1.0008	0.9023	1.0055	1.0093	1.0532
SSP5-8.5	2038	1.0077	1.0044	1.0063	1.0090	1.0111
SSP5-8.5	2039	0.8798	0.7020	0.7041	1.0331	1.0358
SSP5-8.5	2040	0.8765	0.6972	0.7058	1.0356	1.0420
SSP5-8.5	2041	0.9302	0.6964	0.9433	0.9523	1.0355
SSP5-8.5	2042	0.9325	0.6987	0.9425	0.9478	1.0341
SSP5-8.5	2043	0.8508	0.5546	0.6600	1.0737	1.1247
SSP5-8.5	2044	0.7731	0.6292	0.7701	0.7941	0.8779
SSP5-8.5	2045	0.9195	0.7021	0.9462	0.9577	1.0441
SSP5-8.5	2046	0.7661	0.6696	0.6751	0.8624	0.8670
SSP5-8.5	2047	0.7814	0.7728	0.7755	0.7872	0.7904
SSP5-8.5	2048	0.7261	0.7228	0.7246	0.7275	0.7295
SSP5-8.5	2049	0.9439	0.6688	0.6712	1.1192	1.1258
SSP5-8.5	2050	0.7317	0.7240	0.7264	0.7354	0.7375
SSP5-8.5	2051	0.7873	0.7792	0.7814	0.7931	0.7948
SSP5-8.5	2052	0.6787	0.5672	0.5985	0.7750	0.7951
SSP5-8.5	2053	0.7143	0.6912	0.6948	0.7263	0.7301
SSP5-8.5	2054	0.7324	0.5524	0.6041	0.8609	0.9055
SSP5-8.5	2055	0.7664	0.6332	0.6910	0.8251	0.8745
SSP5-8.5	2056	0.7563	0.5966	0.6441	0.8707	0.8774
SSP5-8.5	2057	0.7547	0.5932	0.6528	0.8442	0.8908
SSP5-8.5	2058	0.6718	0.5969	0.6007	0.7254	0.7279
SSP5-8.5	2059	0.6814	0.5772	0.5853	0.7851	0.7892
SSP5-8.5	2060	0.7443	0.5550	0.6471	0.8631	0.8894
SSP5-8.5	2061	0.7084	0.6183	0.7159	0.7343	0.7617
SSP5-8.5	2062	0.8714	0.6879	0.6935	1.0507	1.0576
SSP5-8.5	2063	0.6992	0.5951	0.6197	0.7638	0.7683
SSP5-8.5	2064	0.7617	0.6025	0.6978	0.8192	0.8848
SSP5-8.5	2065	0.6930	0.5743	0.6970	0.7097	0.7381
SSP5-8.5	2066	0.6427	0.6175	0.6195	0.6578	0.6619
SSP5-8.5	2067	0.6899	0.6031	0.6064	0.7491	0.7525

Continue on the next page

**Scenario**	**Year**	**Average**	**Q5**	**Q25**	**Q75**	**Q95**
SSP5-8.5	2068	0.6783	0.6025	0.6067	0.7156	0.7184
SSP5-8.5	2069	0.6383	0.6166	0.6184	0.6570	0.6594
SSP5-8.5	2070	0.6887	0.6037	0.6313	0.7502	0.7532
SSP5-8.5	2071	0.6901	0.6027	0.6958	0.7016	0.7184
SSP5-8.5	2072	0.6450	0.6166	0.6192	0.6588	0.6622
SSP5-8.5	2073	0.6733	0.6034	0.6066	0.7144	0.7176
SSP5-8.5	2074	0.6352	0.5665	0.6168	0.6587	0.6939
SSP5-8.5	2075	0.6756	0.6032	0.6061	0.7386	0.7517
SSP5-8.5	2076	0.6559	0.5529	0.6044	0.7175	0.7393
SSP5-8.5	2077	0.6174	0.5678	0.6176	0.6220	0.6436
SSP5-8.5	2078	0.6177	0.5686	0.6201	0.6236	0.6462
SSP5-8.5	2079	0.6141	0.5678	0.6170	0.6236	0.6459
SSP5-8.5	2080	0.5995	0.4329	0.6186	0.6233	0.6461
SSP5-8.5	2081	0.5979	0.4028	0.6182	0.6223	0.6448
SSP5-8.5	2082	0.6059	0.5301	0.5729	0.6419	0.6457
SSP5-8.5	2083	0.6085	0.5671	0.5702	0.6417	0.6454
SSP5-8.5	2084	0.6123	0.5685	0.6180	0.6232	0.6437
SSP5-8.5	2085	0.5877	0.5872	0.5899	0.5931	0.5956
SSP5-8.5	2086	0.6103	0.5673	0.5706	0.6431	0.6466
SSP5-8.5	2087	0.6144	0.5679	0.6175	0.6240	0.6458
SSP5-8.5	2088	0.6156	0.5677	0.6173	0.6224	0.6444
SSP5-8.5	2089	0.6071	0.4772	0.6186	0.6226	0.6279
SSP5-8.5	2090	0.6148	0.5363	0.6181	0.6221	0.6435
SSP5-8.5	2091	0.6147	0.5668	0.6171	0.6233	0.6452
SSP5-8.5	2092	0.5859	0.4211	0.5700	0.6230	0.6466
SSP5-8.5	2093	0.5746	0.4878	0.5884	0.5928	0.5960
SSP5-8.5	2094	0.5856	0.4069	0.5707	0.6273	0.6453
SSP5-8.5	2095	0.5680	0.3697	0.5697	0.6216	0.6418
SSP5-8.5	2096	0.5733	0.3692	0.5663	0.6241	0.6432
SSP5-8.5	2097	0.5425	0.3397	0.5530	0.5920	0.5960
SSP5-8.5	2098	0.5396	0.3226	0.5396	0.5925	0.5951
SSP5-8.5	2099	0.5629	0.4554	0.5648	0.5879	0.5916
