# Supplementary material for: In hot water: Uncertainties in projecting marine heatwaves impacts on seagrass meadows
Source: PLoS One. 2024 Nov 27;19(11):e0298853. doi: 10.1371/journal.pone.0298853 (PMC11602073; doi:10.1371/journal.pone.0298853)
Supplement: S9 Table — Avg: denotes the average low shoot density ratio per decade. Q25: represents 25th percentile, marking the value below which 25% of the observations fall. Q95: stands for the 95th percentile indicating the value below which 95% of the observations are found. (PDF) [file pone.0298853.s017.pdf]

S9 Table. Low Shoot Density Ratio Across Years for SSP1-1.9 Scenario: This table provides an analysis of the low shoot density states, measured annually within the SSP1-1.9 scenario. Avg: denotes the average low shoot density ratio per decade. **Q25:** represents 25th percentile, marking the value below which 25% of the observations fall. **Q95:** stands for the 95th percentile indicating the value below which 95% of the observations are found.

**Scenario**	**Year**	**Average**	**Q5**	**Q25**	**Q75**	**Q95**
SSP1-1.9	2030	2.4924	2.1604	2.3387	2.3472	4.3735
SSP1-1.9	2031	1.3408	0.9474	0.9889	1.8764	2.4127
SSP1-1.9	2032	1.2430	0.9503	1.0023	1.0044	2.3984
SSP1-1.9	2033	1.0377	0.9998	1.0013	1.0030	1.0041
SSP1-1.9	2034	1.0012	0.9985	1.0001	1.0023	1.0038
SSP1-1.9	2035	2.5169	1.7378	1.7427	3.4763	3.4940
SSP1-1.9	2036	1.0192	0.9987	1.0002	1.0037	1.0056
SSP1-1.9	2037	1.3313	0.9840	0.9977	1.6871	2.2020
SSP1-1.9	2038	2.4685	1.7393	1.7435	3.4851	3.4958
SSP1-1.9	2039	1.6813	0.9495	0.9557	2.3959	2.4340
SSP1-1.9	2040	1.0019	0.9995	1.0009	1.0028	1.0041
SSP1-1.9	2041	3.1381	3.1235	3.1328	3.1433	3.1520
SSP1-1.9	2042	2.4520	1.4663	1.7820	3.1832	4.2465
SSP1-1.9	2043	1.9720	0.9496	0.9849	2.2570	5.7705
SSP1-1.9	2044	2.2728	0.9439	1.2178	3.4085	4.0750
SSP1-1.9	2045	2.5155	1.7179	1.7429	3.4555	3.5539
SSP1-1.9	2046	1.3680	0.9514	0.9851	2.1577	2.4360
SSP1-1.9	2047	2.2141	1.5234	2.0836	2.0915	3.9221
SSP1-1.9	2048	2.1191	2.0705	2.0850	2.0923	2.1017
SSP1-1.9	2049	1.3950	0.9494	0.9699	2.2226	2.4073
SSP1-1.9	2050	2.0899	2.0837	2.0873	2.0926	2.0964
SSP1-1.9	2051	1.7707	0.9478	0.9526	2.4134	3.6268
SSP1-1.9	2052	1.1056	0.9991	1.0005	1.0025	2.7376
SSP1-1.9	2053	1.6044	0.9467	0.9498	2.3934	2.4042
SSP1-1.9	2054	2.4782	1.7329	1.7432	3.4488	3.4960
SSP1-1.9	2055	1.0452	0.9980	0.9999	1.0040	1.0062
SSP1-1.9	2056	1.0018	0.9991	1.0006	1.0031	1.0046
SSP1-1.9	2057	1.0025	1.0005	1.0018	1.0033	1.0043
SSP1-1.9	2058	2.5263	1.7378	1.7433	3.4752	3.4947
SSP1-1.9	2059	5.1275	4.9303	4.9551	5.0428	6.3527
SSP1-1.9	2060	3.2944	2.8694	3.2216	3.2443	4.2127
SSP1-1.9	2061	2.5158	1.7123	1.7227	3.5166	3.5620
SSP1-1.9	2062	1.0021	0.9986	1.0004	1.0041	1.0056
SSP1-1.9	2063	0.9988	0.9959	0.9975	1.0000	1.0019
SSP1-1.9	2064	1.0016	0.9990	1.0006	1.0026	1.0043
SSP1-1.9	2065	1.0668	0.9833	1.0004	1.0030	2.1005
SSP1-1.9	2066	1.0023	1.0001	1.0016	1.0032	1.0043
SSP1-1.9	2067	3.2840	2.6041	3.1324	3.1434	5.4806

Continue on the next page

Scenario	Year	Average	Q5	Q25	Q75	Q95
SSP1-1.9	2068	1.0974	0.9822	1.0008	1.0037	1.9964
SSP1-1.9	2069	2.3690	1.7353	2.0108	2.3535	3.4996
SSP1-1.9	2070	1.2534	0.9498	1.0009	1.0053	2.5343
SSP1-1.9	2071	1.0027	0.9999	1.0018	1.0038	1.0047
SSP1-1.9	2072	1.7720	0.9490	0.9840	2.2692	3.6339
SSP1-1.9	2073	1.5641	0.9490	0.9838	1.9922	3.6254
SSP1-1.9	2074	1.5580	0.9475	1.0000	1.0030	4.2689
SSP1-1.9	2075	2.2436	2.0111	2.1895	2.1967	2.6095
SSP1-1.9	2076	1.2246	0.9499	1.0003	1.0039	2.3935
SSP1-1.9	2077	1.4043	0.9483	0.9839	1.9720	2.5382
SSP1-1.9	2078	2.2430	2.1614	2.1692	2.3626	2.3696
SSP1-1.9	2079	0.9997	0.9968	0.9983	1.0012	1.0026
SSP1-1.9	2080	1.3499	0.9836	0.9854	2.1035	2.1082
SSP1-1.9	2081	3.0871	3.0414	3.0689	3.1050	3.1273
SSP1-1.9	2082	1.7540	0.9524	1.0018	2.7357	4.1510
SSP1-1.9	2083	3.7600	2.4765	2.7239	4.3090	7.0647
SSP1-1.9	2084	1.6489	0.9536	0.9592	2.4099	2.4740
SSP1-1.9	2085	1.0014	0.9985	1.0003	1.0025	1.0037
SSP1-1.9	2086	1.4424	0.9765	0.9813	2.1031	2.3972
SSP1-1.9	2087	1.0015	0.9991	1.0006	1.0024	1.0038
SSP1-1.9	2088	2.3301	1.4720	2.0827	2.0931	4.2346
SSP1-1.9	2089	1.2223	0.9504	1.0019	1.0053	2.5283
SSP1-1.9	2090	0.9991	0.9959	0.9980	1.0003	1.0019
SSP1-1.9	2091	1.2425	0.9680	1.0010	1.0033	2.2243
SSP1-1.9	2092	1.1997	0.9833	1.0010	1.0038	2.1955
SSP1-1.9	2093	1.3978	0.9827	1.0010	1.7486	2.2022
SSP1-1.9	2094	1.0011	0.9983	0.9999	1.0022	1.0041
SSP1-1.9	2095	0.9996	0.9968	0.9984	1.0009	1.0026
SSP1-1.9	2096	1.0021	0.9998	1.0012	1.0030	1.0043
SSP1-1.9	2097	1.3338	0.9481	0.9922	1.3078	2.3959
SSP1-1.9	2098	1.0005	0.9974	0.9991	1.0018	1.0040
SSP1-1.9	2099	1.0023	0.9998	1.0013	1.0034	1.0045
