# Supplementary material for: In hot water: Uncertainties in projecting marine heatwaves impacts on seagrass meadows
Source: PLoS One. 2024 Nov 27;19(11):e0298853. doi: 10.1371/journal.pone.0298853 (PMC11602073; doi:10.1371/journal.pone.0298853)
Supplement: S10 Table — Avg: denotes the average low shoot density ratio per decade. Q25: represents 25th percentile, marking the value below which 25% of the observations fall. Q95: stands for the 95th percentile indicating the value below which 95% of the observations are found. (PDF) [file pone.0298853.s018.pdf]

S10 Table. Low Shoot Density Ratio Across Years for SSP1-2.6 Scenario: This table provides an analysis of the low shoot density states, measured annually within the SSP1-2.6 scenario. Avg: denotes the average low shoot density ratio per decade. **Q25:** represents 25th percentile, marking the value below which 25% of the observations fall. **Q95:** stands for the 95th percentile indicating the value below which 95% of the observations are found.

Scenario	Year	Average	Q5	Q25	Q75	Q95
SSP1-2.6	2030	1.2927	0.9832	1.0013	1.1294	2.1232
SSP1-2.6	2031	1.0017	0.9990	1.0006	1.0028	1.0046
SSP1-2.6	2032	3.1394	3.1203	3.1321	3.1479	3.1536
SSP1-2.6	2033	1.2384	0.9552	1.0034	1.0059	2.4165
SSP1-2.6	2034	1.0408	0.9976	0.9994	1.0020	1.0043
SSP1-2.6	2035	1.0916	0.9842	1.0016	1.0037	1.9890
SSP1-2.6	2036	1.0019	0.9994	1.0009	1.0029	1.0044
SSP1-2.6	2037	1.0513	0.9961	0.9977	1.0008	1.0023
SSP1-2.6	2038	3.1488	3.0921	3.1141	3.1411	3.1575
SSP1-2.6	2039	1.0032	1.0009	1.0021	1.0042	1.0061
SSP1-2.6	2040	3.6730	3.0749	3.3687	3.3786	5.2592
SSP1-2.6	2041	1.0482	1.0023	1.0037	1.0060	1.0090
SSP1-2.6	2042	2.6087	1.9643	1.9715	3.4759	3.5079
SSP1-2.6	2043	3.7760	2.5727	2.5940	5.4446	5.5322
SSP1-2.6	2044	1.0036	0.9995	1.0016	1.0064	1.0082
SSP1-2.6	2045	3.9448	2.6042	2.6172	5.4842	5.5211
SSP1-2.6	2046	1.0048	1.0007	1.0023	1.0074	1.0094
SSP1-2.6	2047	2.0220	0.9479	0.9749	2.3041	3.6410
SSP1-2.6	2048	2.3588	1.7204	1.7503	3.4180	3.5354
SSP1-2.6	2049	3.1484	3.0887	3.1114	3.1845	3.2172
SSP1-2.6	2050	2.3608	2.3555	2.3601	2.3678	2.3717
SSP1-2.6	2051	4.9953	4.9548	4.9778	5.0121	5.0408
SSP1-2.6	2052	3.2402	3.2117	3.2287	3.2511	3.2632
SSP1-2.6	2053	2.5118	1.7190	1.7236	3.5395	3.5541
SSP1-2.6	2054	2.3562	1.7200	2.0803	2.0969	5.2757
SSP1-2.6	2055	1.8597	0.9828	0.9851	2.1076	5.2965
SSP1-2.6	2056	3.0657	1.4540	1.7420	3.4810	6.4299
SSP1-2.6	2057	2.4220	1.7680	2.3095	2.4091	3.6704
SSP1-2.6	2058	1.0028	1.0002	1.0015	1.0039	1.0066
SSP1-2.6	2059	2.5516	1.7401	1.7444	3.4814	5.2283
SSP1-2.6	2060	1.5465	0.9497	0.9695	2.3864	2.6154
SSP1-2.6	2061	1.3277	0.9528	1.0020	1.0053	2.5335
SSP1-2.6	2062	3.2690	2.5971	3.1297	3.1497	5.4958
SSP1-2.6	2063	2.5110	1.7196	2.0817	2.0889	5.2868
SSP1-2.6	2064	3.3699	2.7179	3.1110	3.2106	5.8548
SSP1-2.6	2065	2.8621	1.7021	1.7225	3.5434	5.3312
SSP1-2.6	2066	2.3833	1.4850	1.8866	2.7782	4.2682
SSP1-2.6	2067	3.3730	2.5835	3.1167	3.1986	5.5092

Continue on the next page

**Scenario**	**Year**	**Average**	**Q5**	**Q25**	**Q75**	**Q95**
SSP1-2.6	2068	3.3896	1.2492	2.3038	4.0490	5.9426
SSP1-2.6	2069	3.1872	3.0533	3.1304	3.2371	3.3288
SSP1-2.6	2070	1.5110	1.0028	1.0047	1.7470	3.6313
SSP1-2.6	2071	2.7277	1.7314	1.7417	3.4876	5.2547
SSP1-2.6	2072	3.9929	2.4471	2.7690	4.5755	7.1251
SSP1-2.6	2073	2.3819	2.1738	2.3368	2.4199	2.6149
SSP1-2.6	2074	3.8537	2.5875	2.6079	5.4945	5.5376
SSP1-2.6	2075	2.7060	1.6650	1.7010	3.6148	3.7744
SSP1-2.6	2076	1.1763	0.9999	1.0016	1.0055	2.7336
SSP1-2.6	2077	2.7777	1.5392	1.8674	3.4737	4.6838
SSP1-2.6	2078	3.4259	3.3396	3.3619	3.3957	3.4212
SSP1-2.6	2079	1.8691	0.9528	0.9558	2.4209	4.1851
SSP1-2.6	2080	4.0584	2.3213	2.6153	5.4561	6.6071
SSP1-2.6	2081	1.1870	0.9572	1.0020	1.0075	2.4972
SSP1-2.6	2082	1.1330	0.9827	1.0014	1.0037	2.1094
SSP1-2.6	2083	2.0915	2.0849	2.0889	2.0940	2.0978
SSP1-2.6	2084	1.3968	0.9693	0.9864	1.9212	2.5360
SSP1-2.6	2085	1.0032	1.0013	1.0024	1.0039	1.0049
SSP1-2.6	2086	1.0554	0.9999	1.0012	1.0031	1.6832
SSP1-2.6	2087	1.4096	1.0014	1.0025	1.0049	3.4574
SSP1-2.6	2088	3.9037	2.4683	2.6184	5.4893	5.5401
SSP1-2.6	2089	4.0525	3.0682	3.0992	5.3279	5.4662
SSP1-2.6	2090	1.0062	1.0030	1.0045	1.0084	1.0098
SSP1-2.6	2091	1.1924	0.9997	1.0012	1.0034	2.7338
SSP1-2.6	2092	3.2234	2.7030	3.1288	3.1577	4.3174
SSP1-2.6	2093	2.4730	1.7178	2.0788	2.0889	5.2771
SSP1-2.6	2094	3.3759	2.7091	3.1083	3.1950	5.8643
SSP1-2.6	2095	3.2585	1.2416	1.4697	4.2191	5.9146
SSP1-2.6	2096	2.7565	1.7819	1.9360	3.6058	5.3195
SSP1-2.6	2097	3.6449	2.4586	2.8291	4.3144	5.5568
SSP1-2.6	2098	2.7122	1.6914	1.7262	3.5349	5.2060
SSP1-2.6	2099	3.1193	1.2449	1.4741	4.2269	5.9022
