# Supplementary material for: In hot water: Uncertainties in projecting marine heatwaves impacts on seagrass meadows
Source: PLoS One. 2024 Nov 27;19(11):e0298853. doi: 10.1371/journal.pone.0298853 (PMC11602073; doi:10.1371/journal.pone.0298853)
Supplement: S11 Table — Avg: denotes the average low shoot density ratio per decade. Q25: represents 25th percentile, marking the value below which 25% of the observations fall. Q95: stands for the 95th percentile indicating the value below which 95% of the observations are found. (PDF) [file pone.0298853.s019.pdf]

S11 Table. Low Shoot Density Ratio Across Years for SSP3-7.0 Scenario: This table provides an analysis of the low shoot density states, measured annually within the SSP3-7.0 scenario. Avg: denotes the average low shoot density ratio per decade. **Q25:** represents 25th percentile, marking the value below which 25% of the observations fall. **Q95:** stands for the 95th percentile indicating the value below which 95% of the observations are found.

Scenario	Year	Average	Q5	Q25	Q75	Q95
SSP3-7.0	2030	1.5420	0.9679	0.9724	2.1142	2.1241
SSP3-7.0	2031	1.0764	0.9775	0.9916	0.9949	1.8981
SSP3-7.0	2032	1.0015	0.9992	1.0006	1.0026	1.0039
SSP3-7.0	2033	2.6003	1.9657	1.9731	3.4625	3.4955
SSP3-7.0	2034	1.0005	0.9970	0.9986	1.0028	1.0046
SSP3-7.0	2035	1.0025	1.0002	1.0016	1.0033	1.0046
SSP3-7.0	2036	1.1976	0.9387	0.9950	0.9986	2.3942
SSP3-7.0	2037	1.1134	0.9985	1.0008	1.0032	1.7435
SSP3-7.0	2038	0.9992	0.9957	0.9980	1.0007	1.0028
SSP3-7.0	2039	1.3239	0.9342	0.9871	1.2374	2.3939
SSP3-7.0	2040	1.0597	0.9716	0.9920	0.9951	2.1125
SSP3-7.0	2041	2.2026	1.7366	2.0844	2.0953	3.4731
SSP3-7.0	2042	1.0036	1.0009	1.0025	1.0044	1.0073
SSP3-7.0	2043	1.5348	0.9800	0.9827	2.1085	2.1152
SSP3-7.0	2044	1.0884	0.9812	1.0001	1.0025	2.1079
SSP3-7.0	2045	2.2612	1.7335	2.0811	2.0933	3.4777
SSP3-7.0	2046	1.1580	0.9990	1.0006	1.0030	2.7391
SSP3-7.0	2047	1.4844	0.9436	0.9804	2.2715	2.5676
SSP3-7.0	2048	1.0027	1.0001	1.0018	1.0038	1.0051
SSP3-7.0	2049	1.0517	0.9941	0.9966	0.9999	1.0030
SSP3-7.0	2050	3.3407	3.1075	3.1257	3.1468	4.7161
SSP3-7.0	2051	3.3811	2.5132	3.1552	3.1880	5.4999
SSP3-7.0	2052	1.9589	0.9518	0.9553	2.4190	3.6997
SSP3-7.0	2053	2.8034	1.2533	1.4777	4.1891	5.8415
SSP3-7.0	2054	2.4886	1.8037	2.3129	2.4044	3.6803
SSP3-7.0	2055	1.3724	0.9829	0.9854	2.1053	2.1140
SSP3-7.0	2056	4.2671	3.0756	3.0818	5.2729	6.5964
SSP3-7.0	2057	2.4219	1.6898	1.8043	3.0908	3.6459
SSP3-7.0	2058	2.3902	1.7365	2.0788	2.0950	5.2302
SSP3-7.0	2059	2.3260	1.9928	2.1765	2.3680	2.6207
SSP3-7.0	2060	3.5710	2.5854	3.1218	3.1604	7.1144
SSP3-7.0	2061	3.9519	2.5242	2.6635	5.5988	5.8010
SSP3-7.0	2062	2.4061	0.9571	0.9909	2.3744	5.5571
SSP3-7.0	2063	3.1850	1.3519	1.8994	3.5952	7.8381
SSP3-7.0	2064	4.1584	2.4369	2.6252	5.5348	7.3118
SSP3-7.0	2065	3.5376	2.7768	3.1386	3.2985	5.8159
SSP3-7.0	2066	2.1105	0.9566	0.9883	2.3039	5.4417
SSP3-7.0	2067	3.3549	2.6964	3.1045	3.1754	5.8617

Continue on the next page

**Scenario**	**Year**	**Average**	**Q5**	**Q25**	**Q75**	**Q95**
SSP3-7.0	2068	3.5036	2.5158	3.1604	3.2345	5.6387
SSP3-7.0	2069	3.5293	3.1373	3.1879	3.2109	4.8534
SSP3-7.0	2070	4.9781	2.4142	2.6825	7.0586	8.2746
SSP3-7.0	2071	5.7378	4.8338	4.9204	6.2081	7.5052
SSP3-7.0	2072	5.6228	4.9774	5.2222	5.3042	7.4893
SSP3-7.0	2073	2.6026	1.8332	2.2608	2.5183	3.8519
SSP3-7.0	2074	4.0352	2.5982	2.8105	4.6426	7.2011
SSP3-7.0	2075	5.3431	3.5694	3.9263	7.1630	8.2884
SSP3-7.0	2076	5.9949	5.9423	5.9706	6.0184	6.0408
SSP3-7.0	2077	4.2481	2.7851	2.7983	5.7635	5.8006
SSP3-7.0	2078	6.4414	6.2945	6.3269	6.5159	6.5627
SSP3-7.0	2079	6.4192	5.9941	6.0201	7.1997	7.2397
SSP3-7.0	2080	4.6862	2.5865	2.7190	5.8167	8.0051
SSP3-7.0	2081	5.3143	4.8758	5.1178	5.3125	6.2566
SSP3-7.0	2082	5.6996	5.1880	5.2301	6.5290	6.6967
SSP3-7.0	2083	5.9434	4.9986	5.0452	7.4743	7.5434
SSP3-7.0	2084	6.7651	6.1617	6.2047	7.4840	7.5351
SSP3-7.0	2085	6.5289	5.9679	5.9951	7.1740	7.2083
SSP3-7.0	2086	5.8499	5.0461	5.0807	6.2224	7.5050
SSP3-7.0	2087	3.9541	2.6368	3.0193	5.6033	7.2763
SSP3-7.0	2088	6.6586	6.0238	6.0733	7.5100	7.5815
SSP3-7.0	2089	5.1332	3.5288	3.5700	7.8333	7.9029
SSP3-7.0	2090	4.7782	2.4769	2.6323	7.1012	8.2996
SSP3-7.0	2091	6.0617	4.9812	5.0470	7.4741	7.6550
SSP3-7.0	2092	5.8885	3.6877	3.9484	7.4009	8.3660
SSP3-7.0	2093	7.2857	6.8915	7.1956	7.2566	8.4157
SSP3-7.0	2094	7.2711	7.2144	7.2492	7.2915	7.3314
SSP3-7.0	2095	7.3395	6.9139	7.2246	7.2876	8.5027
SSP3-7.0	2096	7.2736	7.1853	7.2204	7.2715	7.3182
SSP3-7.0	2097	7.2556	7.1852	7.2263	7.2841	7.3299
SSP3-7.0	2098	7.2091	7.1496	7.1870	7.2309	7.2709
SSP3-7.0	2099	7.2052	7.1492	7.1826	7.2275	7.2653
