# Supplementary material for: In hot water: Uncertainties in projecting marine heatwaves impacts on seagrass meadows
Source: PLoS One. 2024 Nov 27;19(11):e0298853. doi: 10.1371/journal.pone.0298853 (PMC11602073; doi:10.1371/journal.pone.0298853)
Supplement: S12 Table — Avg: denotes the average low shoot density ratio per decade. Q25: represents 25th percentile, marking the value below which 25% of the observations fall. Q95: stands for the 95th percentile indicating the value below which 95% of the observations are found. (PDF) [file pone.0298853.s020.pdf]

S12 Table. Low Shoot Density Ratio Across Years for SSP5-8.5 Scenario: This table provides an analysis of the low shoot density states, measured annually within the SSP5-8.5 scenario. Avg: denotes the average low shoot density ratio per decade. **Q25:** represents 25th percentile, marking the value below which 25% of the observations fall. **Q95:** stands for the 95th percentile indicating the value below which 95% of the observations are found.

**Scenario**	**Year**	**Average**	**Q5**	**Q25**	**Q75**	**Q95**
SSP5-8.5	2030	2.6452	1.4669	1.7913	3.1884	5.9833
SSP5-8.5	2031	1.5114	0.9792	0.9840	2.1008	2.4732
SSP5-8.5	2032	2.3969	1.7362	1.7446	3.4446	3.4796
SSP5-8.5	2033	1.7073	0.9480	0.9843	1.9910	5.3320
SSP5-8.5	2034	3.1911	2.6914	3.1149	3.1470	4.2986
SSP5-8.5	2035	1.0043	1.0016	1.0032	1.0053	1.0076
SSP5-8.5	2036	1.0142	0.9976	0.9992	1.0016	1.0035
SSP5-8.5	2037	1.1207	0.9807	0.9999	1.0025	2.1093
SSP5-8.5	2038	1.0031	1.0010	1.0023	1.0040	1.0051
SSP5-8.5	2039	2.5410	1.7393	1.7434	3.4841	3.4975
SSP5-8.5	2040	2.5809	1.7188	1.7397	3.4888	3.5664
SSP5-8.5	2041	2.2079	1.7240	2.0823	2.0972	3.5408
SSP5-8.5	2042	2.1727	1.7347	2.0838	2.0933	3.4887
SSP5-8.5	2043	3.0133	1.2542	1.4754	4.2101	5.8448
SSP5-8.5	2044	3.4671	2.5646	3.0886	3.2899	5.5790
SSP5-8.5	2045	2.3669	1.7228	2.0742	2.0952	3.5528
SSP5-8.5	2046	4.2179	3.0694	3.0889	5.2793	5.3602
SSP5-8.5	2047	3.2128	3.1437	3.1596	3.2652	3.2842
SSP5-8.5	2048	5.0551	5.0218	5.0377	5.0710	5.0896
SSP5-8.5	2049	2.6426	1.8985	1.9039	3.7267	3.7461
SSP5-8.5	2050	5.0417	4.9662	4.9895	5.0954	5.1243
SSP5-8.5	2051	4.0294	3.2347	3.2429	4.7844	4.7995
SSP5-8.5	2052	6.0172	4.6513	4.7670	6.8212	7.4328
SSP5-8.5	2053	5.6220	5.1277	5.1773	6.5363	6.6038
SSP5-8.5	2054	4.4178	2.5237	2.6580	5.6953	6.6826
SSP5-8.5	2055	3.7872	2.6307	2.9197	4.2576	7.0041
SSP5-8.5	2056	4.5363	2.5373	2.6593	6.8852	7.2971
SSP5-8.5	2057	4.2102	2.5195	2.8102	5.6759	7.3224
SSP5-8.5	2058	6.6807	5.9674	6.0243	7.4776	7.5298
SSP5-8.5	2059	5.1216	3.4840	3.5000	6.3699	7.5521
SSP5-8.5	2060	4.7316	2.4759	2.8001	5.7934	8.2615
SSP5-8.5	2061	5.4082	4.8552	5.1114	5.3454	6.2758
SSP5-8.5	2062	3.2766	1.6829	1.6970	5.1664	5.4062
SSP5-8.5	2063	5.5532	4.7660	4.8191	6.2371	7.5459
SSP5-8.5	2064	3.9363	2.6318	3.0155	4.2298	7.3386
SSP5-8.5	2065	6.5887	5.7916	6.3227	6.5246	8.1492
SSP5-8.5	2066	6.4445	5.9469	5.9798	7.1623	7.1981
SSP5-8.5	2067	5.9005	5.0308	5.0660	7.3617	7.5086

Continue on the next page

Scenario	Year	Average	Q5	Q25	Q75	Q95
SSP5-8.5	2068	6.7400	6.1916	6.2439	7.4882	7.5592
SSP5-8.5	2069	6.5604	5.9674	5.9889	7.1716	7.2063
SSP5-8.5	2070	5.7779	5.0440	5.0806	6.2383	7.4286
SSP5-8.5	2071	6.5723	6.1191	6.4575	6.5508	7.4978
SSP5-8.5	2072	6.3961	5.9562	5.9922	7.1827	7.2323
SSP5-8.5	2073	6.8405	6.2622	6.3104	7.5503	7.6252
SSP5-8.5	2074	6.7477	5.8302	6.0241	7.2489	8.4610
SSP5-8.5	2075	6.2190	5.0456	5.1074	7.3847	7.5167
SSP5-8.5	2076	7.1199	5.9579	6.2822	7.6070	8.8219
SSP5-8.5	2077	7.2876	6.8434	7.1742	7.2476	8.4210
SSP5-8.5	2078	7.3658	6.8754	7.2035	7.2761	8.5110
SSP5-8.5	2079	7.4652	6.8954	7.2125	7.3452	8.5358
SSP5-8.5	2080	7.6866	6.9599	7.2891	7.3752	10.5487
SSP5-8.5	2081	7.7061	6.9547	7.2841	7.3584	10.8764
SSP5-8.5	2082	7.7094	6.8528	7.0460	8.5415	9.0069
SSP5-8.5	2083	7.6639	6.9158	6.9716	8.5024	8.5775
SSP5-8.5	2084	7.4846	6.9544	7.2794	7.3741	8.5387
SSP5-8.5	2085	8.3736	8.2122	8.2615	8.3297	8.4030
SSP5-8.5	2086	7.6963	6.9712	7.0292	8.5871	8.6773
SSP5-8.5	2087	7.5863	7.0007	7.3271	7.4572	8.6616
SSP5-8.5	2088	7.4437	6.9319	7.2517	7.3569	8.5660
SSP5-8.5	2089	7.5406	7.1996	7.2817	7.3629	9.4535
SSP5-8.5	2090	7.4398	6.9469	7.2778	7.3585	9.0152
SSP5-8.5	2091	7.4437	6.8746	7.2204	7.3473	8.5544
SSP5-8.5	2092	7.9549	6.9226	7.1146	8.5176	10.7655
SSP5-8.5	2093	8.6027	8.0181	8.2147	8.3307	10.5108
SSP5-8.5	2094	8.0664	7.0104	7.2125	8.6414	10.9803
SSP5-8.5	2095	8.3518	7.0078	7.3521	9.0201	11.4443
SSP5-8.5	2096	8.2205	6.8914	7.2162	8.8991	11.4509
SSP5-8.5	2097	9.0732	8.0568	8.2757	9.2847	12.1829
SSP5-8.5	2098	9.1908	8.1210	8.3165	9.4753	12.4719
SSP5-8.5	2099	9.0191	8.2260	8.4888	8.9123	11.3111
