# Supplementary material for: In hot water: Uncertainties in projecting marine heatwaves impacts on seagrass meadows
Source: PLoS One. 2024 Nov 27;19(11):e0298853. doi: 10.1371/journal.pone.0298853 (PMC11602073; doi:10.1371/journal.pone.0298853)
Supplement: S13 Table — Avg: denotes the average zero shoot density ratio per decade. Q25: represents 25th percentile, marking the value below which 25% of the observations fall. Q95: stands for the 95th percentile indicating the value below which 95% of the observations are found. (PDF) [file pone.0298853.s021.pdf]

S13 Table. Zero Shoot Density Ratio Across Years for SSP1-1.9 Scenario: This table provides an analysis of the zero shoot density states, measured annually within the SSP1-1.9 scenario. Avg: denotes the average zero shoot density ratio per decade. **Q25:** represents 25th percentile, marking the value below which 25% of the observations fall. **Q95:** stands for the 95th percentile indicating the value below which 95% of the observations are found.

Scenario	Year	Average	Q5	Q25	Q75	Q95
SSP1-1.9	2030	1.0696	0.8189	0.8227	0.8291	2.7824
SSP1-1.9	2031	0.9451	0.5460	0.7389	0.9907	1.2170
SSP1-1.9	2032	0.9632	0.5900	0.9896	0.9947	1.2317
SSP1-1.9	2033	0.9810	0.9780	0.9827	0.9867	0.9890
SSP1-1.9	2034	0.9893	0.9842	0.9873	0.9915	0.9941
SSP1-1.9	2035	1.3838	0.9253	0.9285	2.0374	2.0416
SSP1-1.9	2036	1.0011	0.9868	0.9898	1.0028	1.0062
SSP1-1.9	2037	0.9775	0.7398	0.8712	1.0144	1.3175
SSP1-1.9	2038	1.3283	0.9245	0.9271	2.0369	2.0425
SSP1-1.9	2039	0.9575	0.5887	0.5915	1.2217	1.2800
SSP1-1.9	2040	0.9883	0.9829	0.9858	0.9904	0.9932
SSP1-1.9	2041	2.0382	2.0327	2.0366	2.0403	2.0427
SSP1-1.9	2042	1.2583	0.9482	0.9515	1.5139	2.5438
SSP1-1.9	2043	1.1339	0.5901	0.7418	1.4164	2.8135
SSP1-1.9	2044	1.2746	0.5817	0.5930	1.9171	2.3490
SSP1-1.9	2045	1.3602	0.9231	0.9288	2.0338	2.1195
SSP1-1.9	2046	0.9868	0.5898	0.6999	1.2297	1.2866
SSP1-1.9	2047	1.0295	0.9241	0.9294	0.9355	1.9090
SSP1-1.9	2048	0.9650	0.9243	0.9272	0.9316	0.9627
SSP1-1.9	2049	0.9482	0.5891	0.6440	1.2227	1.2416
SSP1-1.9	2050	0.9301	0.9239	0.9273	0.9327	0.9347
SSP1-1.9	2051	1.0204	0.5905	0.5961	1.4164	2.1148
SSP1-1.9	2052	1.0255	0.9872	0.9895	0.9942	1.5420
SSP1-1.9	2053	0.9555	0.5875	0.5916	1.2253	1.2296
SSP1-1.9	2054	1.3484	0.9205	0.9275	2.0266	2.0441
SSP1-1.9	2055	0.9952	0.9873	0.9917	1.0053	1.0093
SSP1-1.9	2056	0.9924	0.9873	0.9902	0.9947	0.9979
SSP1-1.9	2057	0.9876	0.9834	0.9860	0.9892	0.9917
SSP1-1.9	2058	1.3929	0.9241	0.9275	2.0342	2.0392
SSP1-1.9	2059	3.1579	3.0297	3.0350	3.1087	4.0273
SSP1-1.9	2060	2.3953	2.2509	2.2567	2.2687	3.6013
SSP1-1.9	2061	1.4270	0.9471	0.9523	2.1295	2.1421
SSP1-1.9	2062	0.9883	0.9784	0.9822	0.9961	0.9994
SSP1-1.9	2063	0.9888	0.9839	0.9865	0.9909	0.9942
SSP1-1.9	2064	0.9933	0.9886	0.9914	0.9952	0.9979
SSP1-1.9	2065	0.9876	0.7447	0.9890	0.9936	1.1840
SSP1-1.9	2066	0.9893	0.9849	0.9875	0.9911	0.9937
SSP1-1.9	2067	2.1176	2.0334	2.0369	2.0407	3.0311

Continue on the next page

**Scenario**	**Year**	**Average**	**Q5**	**Q25**	**Q75**	**Q95**
SSP1-1.9	2068	0.9994	0.7524	1.0012	1.0068	1.0338
SSP1-1.9	2069	1.2158	0.9249	0.9286	1.0716	2.0446
SSP1-1.9	2070	0.9732	0.5898	0.9867	1.0011	1.4194
SSP1-1.9	2071	0.9914	0.9851	0.9896	0.9936	0.9957
SSP1-1.9	2072	1.0719	0.5896	0.7401	1.4142	2.1126
SSP1-1.9	2073	1.0185	0.5880	0.7393	1.1832	2.1049
SSP1-1.9	2074	1.0829	0.5874	0.9830	0.9892	1.9664
SSP1-1.9	2075	1.1411	0.8412	0.8758	0.8802	2.3067
SSP1-1.9	2076	0.9629	0.5855	0.9825	0.9908	1.2190
SSP1-1.9	2077	0.9699	0.5889	0.7401	1.0383	1.4135
SSP1-1.9	2078	0.8546	0.8055	0.8201	0.8748	0.8773
SSP1-1.9	2079	0.9895	0.9837	0.9873	0.9919	0.9946
SSP1-1.9	2080	0.9722	0.7403	0.7433	1.1811	1.1842
SSP1-1.9	2081	2.0334	2.0252	2.0296	2.0371	2.0418
SSP1-1.9	2082	1.1994	0.6160	1.0025	1.5549	1.8418
SSP1-1.9	2083	2.7992	2.0340	2.0407	3.6303	4.7184
SSP1-1.9	2084	1.0044	0.5778	0.6223	1.2936	1.4005
SSP1-1.9	2085	0.9880	0.9824	0.9854	0.9905	0.9929
SSP1-1.9	2086	0.9968	0.7328	0.7407	1.1812	1.4160
SSP1-1.9	2087	0.9865	0.9811	0.9846	0.9881	0.9910
SSP1-1.9	2088	1.1354	0.9267	0.9298	0.9359	2.4655
SSP1-1.9	2089	0.9744	0.5913	0.9883	0.9983	1.4190
SSP1-1.9	2090	0.9880	0.9815	0.9859	0.9901	0.9933
SSP1-1.9	2091	0.9618	0.6391	0.9820	0.9871	1.2206
SSP1-1.9	2092	0.9889	0.7364	0.9808	0.9867	1.3123
SSP1-1.9	2093	1.0132	0.7386	0.8977	1.0568	1.3217
SSP1-1.9	2094	0.9914	0.9860	0.9886	0.9931	0.9987
SSP1-1.9	2095	0.9911	0.9862	0.9890	0.9931	0.9960
SSP1-1.9	2096	0.9908	0.9868	0.9893	0.9924	0.9952
SSP1-1.9	2097	0.9493	0.5877	0.8990	1.0494	1.2266
SSP1-1.9	2098	0.9924	0.9865	0.9900	0.9949	0.9981
SSP1-1.9	2099	0.9921	0.9875	0.9902	0.9942	0.9967
