# Supplementary material for: In hot water: Uncertainties in projecting marine heatwaves impacts on seagrass meadows
Source: PLoS One. 2024 Nov 27;19(11):e0298853. doi: 10.1371/journal.pone.0298853 (PMC11602073; doi:10.1371/journal.pone.0298853)
Supplement: S14 Table — Avg: denotes the average zero shoot density ratio per decade. Q25: represents 25th percentile, marking the value below which 25% of the observations fall. Q95: stands for the 95th percentile indicating the value below which 95% of the observations are found. (PDF) [file pone.0298853.s022.pdf]

S14 Table. Zero Shoot Density Ratio Across Years for SSP1-2.6 Scenario: This table provides an analysis of the zero shoot density states, measured annually within the SSP1-2.6 scenario. Avg: denotes the average zero shoot density ratio per decade. **Q25:** represents 25th percentile, marking the value below which 25% of the observations fall. **Q95:** stands for the 95th percentile indicating the value below which 95% of the observations are found.

Scenario	Year	Average	Q5	Q25	Q75	Q95
SSP1-2.6	2030	0.9837	0.7388	0.9727	0.9917	1.1835
SSP1-2.6	2031	0.9914	0.9863	0.9893	0.9933	0.9968
SSP1-2.6	2032	2.0396	2.0343	2.0375	2.0421	2.0446
SSP1-2.6	2033	0.9878	0.6185	0.9990	1.0043	1.2875
SSP1-2.6	2034	0.9851	0.9839	0.9880	0.9930	0.9965
SSP1-2.6	2035	0.9985	0.7454	0.9906	0.9946	1.1841
SSP1-2.6	2036	0.9878	0.9825	0.9857	0.9900	0.9929
SSP1-2.6	2037	1.0061	0.9844	0.9872	0.9923	0.9952
SSP1-2.6	2038	2.0867	2.0318	2.0362	2.0420	2.0460
SSP1-2.6	2039	1.0033	0.9988	1.0012	1.0050	1.0082
SSP1-2.6	2040	2.1247	1.9076	1.9103	1.9153	3.0351
SSP1-2.6	2041	1.0088	1.0024	1.0066	1.0115	1.0232
SSP1-2.6	2042	1.2515	0.8186	0.8221	2.0405	2.0456
SSP1-2.6	2043	2.4052	2.0202	2.0243	3.0887	3.1112
SSP1-2.6	2044	1.0101	0.9989	1.0026	1.0217	1.0254
SSP1-2.6	2045	2.4836	2.0434	2.0464	3.0392	3.0438
SSP1-2.6	2046	1.0162	1.0039	1.0068	1.0270	1.0315
SSP1-2.6	2047	1.1786	0.5884	0.7001	1.4133	2.1117
SSP1-2.6	2048	1.2239	0.9201	0.9270	2.0157	2.1103
SSP1-2.6	2049	2.0632	2.0370	2.0410	2.1139	2.1253
SSP1-2.6	2050	0.8968	0.8884	0.8917	0.8978	0.9020
SSP1-2.6	2051	3.0216	3.0155	3.0196	3.0237	3.0265
SSP1-2.6	2052	2.2686	2.2554	2.2628	2.2744	2.2811
SSP1-2.6	2053	1.4118	0.9490	0.9529	2.1281	2.1329
SSP1-2.6	2054	1.0982	0.9246	0.9284	0.9518	2.6073
SSP1-2.6	2055	1.0861	0.7105	0.7396	1.1768	2.3707
SSP1-2.6	2056	1.7176	0.9235	0.9291	2.0366	3.3911
SSP1-2.6	2057	1.2215	0.8248	0.8281	0.9908	2.8307
SSP1-2.6	2058	0.9924	0.9862	0.9885	0.9935	1.0067
SSP1-2.6	2059	1.3003	0.9245	0.9271	2.0370	2.5914
SSP1-2.6	2060	1.0168	0.5889	0.6166	1.2263	2.4235
SSP1-2.6	2061	1.0116	0.5899	0.9848	0.9931	1.4193
SSP1-2.6	2062	2.1138	2.0257	2.0400	2.0462	3.0356
SSP1-2.6	2063	1.2346	0.9492	0.9521	0.9572	2.6768
SSP1-2.6	2064	2.3183	2.0385	2.0417	2.1238	4.3243
SSP1-2.6	2065	1.6409	0.9524	0.9565	2.1324	2.8935
SSP1-2.6	2066	1.1910	0.9216	0.9336	1.3647	2.2531
SSP1-2.6	2067	2.1895	2.0406	2.0441	2.1041	3.1314

Continue on the next page

Scenario	Year	Average	Q5	Q25	Q75	Q95
SSP1-2.6	2068	2.0116	0.9482	1.3746	2.5528	3.1139
SSP1-2.6	2069	2.1539	2.0479	2.1084	2.1795	2.2752
SSP1-2.6	2070	1.0941	0.9086	0.9115	1.1645	1.9308
SSP1-2.6	2071	1.4658	0.9280	0.9325	2.0471	2.7490
SSP1-2.6	2072	2.6631	2.0333	2.0387	2.7911	4.8335
SSP1-2.6	2073	1.2017	0.8922	0.8965	1.0180	2.2754
SSP1-2.6	2074	2.4178	2.0189	2.0230	3.0175	3.0515
SSP1-2.6	2075	1.5926	0.9518	0.9557	2.1686	2.3194
SSP1-2.6	2076	1.0481	0.9810	0.9842	0.9991	1.5621
SSP1-2.6	2077	1.6634	0.8515	0.8792	2.0491	3.6366
SSP1-2.6	2078	1.9574	1.9040	1.9099	1.9342	1.9517
SSP1-2.6	2079	1.1038	0.6266	0.6286	1.3105	2.0603
SSP1-2.6	2080	2.6861	2.0199	2.0278	3.0308	4.3348
SSP1-2.6	2081	1.0159	0.6252	1.0012	1.0219	1.4838
SSP1-2.6	2082	0.9840	0.7387	0.9884	0.9931	1.1858
SSP1-2.6	2083	0.9276	0.9234	0.9258	0.9295	0.9323
SSP1-2.6	2084	1.0114	0.5971	0.7908	0.9929	1.4217
SSP1-2.6	2085	0.9914	0.9865	0.9895	0.9926	0.9950
SSP1-2.6	2086	0.9897	0.8963	0.9843	0.9881	1.1462
SSP1-2.6	2087	1.0833	0.8975	0.9875	0.9918	1.7129
SSP1-2.6	2088	2.6240	2.0416	2.0459	3.0554	4.0355
SSP1-2.6	2089	2.4244	1.9305	1.9358	3.1144	3.2340
SSP1-2.6	2090	1.0165	1.0065	1.0099	1.0253	1.0290
SSP1-2.6	2091	1.0508	0.9848	0.9869	0.9916	1.5522
SSP1-2.6	2092	2.2251	2.0442	2.0476	2.0538	3.6434
SSP1-2.6	2093	1.2083	0.9481	0.9513	0.9578	2.6938
SSP1-2.6	2094	2.3166	2.0445	2.0482	2.1289	4.3263
SSP1-2.6	2095	1.9437	0.9473	0.9536	2.5449	3.1608
SSP1-2.6	2096	1.4133	0.8246	0.8432	2.1521	2.7851
SSP1-2.6	2097	2.4200	2.0209	2.0256	2.7029	4.0312
SSP1-2.6	2098	1.5311	0.9477	0.9518	2.1213	2.8115
SSP1-2.6	2099	1.8399	0.9304	0.9412	2.4854	3.1263
