# Supplementary material for: In hot water: Uncertainties in projecting marine heatwaves impacts on seagrass meadows
Source: PLoS One. 2024 Nov 27;19(11):e0298853. doi: 10.1371/journal.pone.0298853 (PMC11602073; doi:10.1371/journal.pone.0298853)
Supplement: S15 Table — Avg: denotes the average zero shoot density ratio per decade. Q25: represents 25th percentile, marking the value below which 25% of the observations fall. Q95: stands for the 95th percentile indicating the value below which 95% of the observations are found. (PDF) [file pone.0298853.s023.pdf]

S15 Table. Zero Shoot Density Ratio Across Years for SSP3-7.0 Scenario: This table provides an analysis of the zero shoot density states, measured annually within the SSP3-7.0 scenario. Avg: denotes the average zero shoot density ratio per decade. **Q25:** represents 25th percentile, marking the value below which 25% of the observations fall. **Q95:** stands for the 95th percentile indicating the value below which 95% of the observations are found.

**Scenario**	**Year**	**Average**	**Q5**	**Q25**	**Q75**	**Q95**
SSP3-7.0	2030	0.9914	0.7225	0.7282	1.1733	1.1789
SSP3-7.0	2031	0.9672	0.7227	0.9815	0.9866	0.9942
SSP3-7.0	2032	0.9876	0.9827	0.9860	0.9897	0.9924
SSP3-7.0	2033	1.2349	0.8164	0.8193	2.0370	2.0427
SSP3-7.0	2034	0.9892	0.9797	0.9826	0.9985	1.0024
SSP3-7.0	2035	0.9914	0.9868	0.9897	0.9930	0.9960
SSP3-7.0	2036	0.9622	0.5858	0.9837	0.9895	1.2239
SSP3-7.0	2037	0.9899	0.8854	0.9847	0.9898	1.1572
SSP3-7.0	2038	0.9902	0.9841	0.9876	0.9926	0.9981
SSP3-7.0	2039	0.9458	0.5855	0.9164	0.9875	1.2232
SSP3-7.0	2040	0.9784	0.7275	0.9797	0.9850	1.1747
SSP3-7.0	2041	1.0491	0.9207	0.9249	0.9306	2.0374
SSP3-7.0	2042	0.9973	0.9917	0.9942	0.9979	1.0099
SSP3-7.0	2043	0.9587	0.7344	0.7385	1.1781	1.1825
SSP3-7.0	2044	0.9851	0.7382	0.9876	0.9920	1.1829
SSP3-7.0	2045	1.1207	0.9247	0.9292	0.9362	2.0475
SSP3-7.0	2046	1.0445	0.9890	0.9918	0.9964	1.5525
SSP3-7.0	2047	0.9748	0.5334	0.7362	0.9907	1.4174
SSP3-7.0	2048	0.9945	0.9886	0.9923	0.9965	0.9994
SSP3-7.0	2049	0.9801	0.9780	0.9839	0.9890	0.9928
SSP3-7.0	2050	2.1337	2.0346	2.0399	2.0449	2.7325
SSP3-7.0	2051	2.3687	2.1157	2.1200	2.1278	4.0262
SSP3-7.0	2052	1.1012	0.6169	0.6202	1.3030	2.2470
SSP3-7.0	2053	1.5289	0.9241	0.9294	2.4523	3.0409
SSP3-7.0	2054	1.0907	0.8226	0.8258	0.9438	2.2066
SSP3-7.0	2055	0.9700	0.7360	0.7408	1.1771	1.1891
SSP3-7.0	2056	2.5213	1.9076	1.9105	3.0311	3.8115
SSP3-7.0	2057	1.3595	0.9618	0.9665	1.8395	2.2671
SSP3-7.0	2058	1.1509	0.9309	0.9350	0.9585	2.6128
SSP3-7.0	2059	1.0231	0.8262	0.8289	0.8836	2.3031
SSP3-7.0	2060	2.2959	2.0256	2.0295	2.0382	3.7595
SSP3-7.0	2061	2.6377	2.1254	2.1309	3.1663	4.0843
SSP3-7.0	2062	1.4183	0.6216	0.7621	1.5633	2.9228
SSP3-7.0	2063	1.8305	0.8181	0.8330	2.1310	4.5700
SSP3-7.0	2064	2.7635	2.0290	2.0508	3.1464	4.8128
SSP3-7.0	2065	2.5202	2.1299	2.1368	2.2854	4.3399
SSP3-7.0	2066	1.2676	0.6160	0.7573	1.4842	2.7800
SSP3-7.0	2067	2.2598	2.0284	2.0444	2.1060	4.3368

Continue on the next page

Scenario	Year	Average	Q5	Q25	Q75	Q95
SSP3-7.0	2068	2.4117	2.1255	2.1299	2.1525	4.0425
SSP3-7.0	2069	2.3126	2.1347	2.1383	2.1456	2.9900
SSP3-7.0	2070	3.3312	2.1280	2.1323	3.9883	5.3746
SSP3-7.0	2071	3.9211	3.1268	3.1937	4.3445	5.3789
SSP3-7.0	2072	3.6261	3.2441	3.2502	3.3011	5.3000
SSP3-7.0	2073	1.5443	1.0372	1.0421	1.6825	2.9453
SSP3-7.0	2074	2.5436	2.0384	2.0414	2.7660	3.9001
SSP3-7.0	2075	3.5903	2.8566	2.8679	4.1808	5.3915
SSP3-7.0	2076	4.3477	4.3345	4.3411	4.3534	4.3573
SSP3-7.0	2077	2.8118	2.3417	2.3496	3.3063	3.3104
SSP3-7.0	2078	4.1777	4.0613	4.0737	4.2149	4.2356
SSP3-7.0	2079	4.6847	4.3251	4.3291	5.3964	5.4158
SSP3-7.0	2080	3.3370	2.3561	2.3664	4.0861	5.3166
SSP3-7.0	2081	3.3678	3.1324	3.1385	3.3007	4.3370
SSP3-7.0	2082	3.5916	3.2470	3.2514	4.2220	4.3176
SSP3-7.0	2083	3.9434	3.2538	3.2584	5.3035	5.3986
SSP3-7.0	2084	4.6693	4.2266	4.2406	5.3954	5.4119
SSP3-7.0	2085	4.8141	4.3338	4.3377	5.3943	5.4077
SSP3-7.0	2086	3.9584	3.3023	3.3072	4.3325	5.3814
SSP3-7.0	2087	2.8574	2.2818	2.2924	3.5699	4.9015
SSP3-7.0	2088	4.4848	4.0854	4.0995	5.3954	5.4234
SSP3-7.0	2089	4.2021	3.6084	3.6161	5.1921	5.4241
SSP3-7.0	2090	3.4387	2.2138	2.2233	4.8079	5.3337
SSP3-7.0	2091	4.0265	3.1279	3.1801	5.3315	5.4406
SSP3-7.0	2092	4.1122	3.0527	3.0870	5.0923	5.4376
SSP3-7.0	2093	5.4939	5.4012	5.4128	5.4356	6.4310
SSP3-7.0	2094	5.4316	5.4136	5.4252	5.4378	5.4486
SSP3-7.0	2095	5.5231	5.4082	5.4199	5.4375	6.4927
SSP3-7.0	2096	5.4573	5.4068	5.4175	5.4324	5.4493
SSP3-7.0	2097	5.4242	5.4023	5.4149	5.4319	5.4454
SSP3-7.0	2098	5.4111	5.3882	5.4044	5.4191	5.4289
SSP3-7.0	2099	5.4052	5.3829	5.3991	5.4129	5.4249
