# Supplementary material for: In hot water: Uncertainties in projecting marine heatwaves impacts on seagrass meadows
Source: PLoS One. 2024 Nov 27;19(11):e0298853. doi: 10.1371/journal.pone.0298853 (PMC11602073; doi:10.1371/journal.pone.0298853)
Supplement: S16 Table — Avg: denotes the average zero shoot density ratio per decade. Q25: represents 25th percentile, marking the value below which 25% of the observations fall. Q95: stands for the 95th percentile indicating the value below which 95% of the observations are found. (PDF) [file pone.0298853.s024.pdf]

S16 Table. Zero Shoot Density Ratio Across Years for SSP5-8.5 Scenario: This table provides an analysis of the zero shoot density states, measured annually within the SSP5-8.5 scenario. Avg: denotes the average zero shoot density ratio per decade. **Q25:** represents 25th percentile, marking the value below which 25% of the observations fall. **Q95:** stands for the 95th percentile indicating the value below which 95% of the observations are found.

Scenario	Year	Average	Q5	Q25	Q75	Q95
SSP5-8.5	2030	1.3565	0.9249	0.9290	1.4946	3.0805
SSP5-8.5	2031	0.9733	0.5535	0.7450	1.1870	1.2100
SSP5-8.5	2032	1.2600	0.9219	0.9255	2.0296	2.0382
SSP5-8.5	2033	1.0775	0.5881	0.7414	1.1847	2.6879
SSP5-8.5	2034	2.1713	2.0217	2.0380	2.0454	3.6210
SSP5-8.5	2035	1.0068	1.0016	1.0041	1.0083	1.0181
SSP5-8.5	2036	0.9912	0.9848	0.9878	0.9919	0.9951
SSP5-8.5	2037	0.9843	0.7387	0.9880	0.9929	1.1851
SSP5-8.5	2038	0.9927	0.9884	0.9907	0.9945	0.9967
SSP5-8.5	2039	1.4072	0.9264	0.9288	2.0404	2.0453
SSP5-8.5	2040	1.4651	0.9291	0.9328	2.0476	2.1271
SSP5-8.5	2041	1.0673	0.9278	0.9311	0.9536	2.1194
SSP5-8.5	2042	1.0232	0.9254	0.9292	0.9348	2.0451
SSP5-8.5	2043	1.6942	0.9247	0.9302	2.4644	3.0308
SSP5-8.5	2044	2.2788	2.0424	2.0474	2.1925	3.2130
SSP5-8.5	2045	1.2221	0.9464	0.9492	0.9828	2.1274
SSP5-8.5	2046	2.5251	1.9124	1.9164	3.0458	3.1277
SSP5-8.5	2047	2.2115	2.1577	2.1626	2.2568	2.2703
SSP5-8.5	2048	3.1220	3.1158	3.1191	3.1247	3.1286
SSP5-8.5	2049	1.3958	1.0132	1.0164	2.2673	2.2772
SSP5-8.5	2050	3.0451	3.0109	3.0150	3.1083	3.1168
SSP5-8.5	2051	2.6454	2.2655	2.2703	3.0070	3.0187
SSP5-8.5	2052	4.0568	3.1206	3.1293	4.7946	5.2529
SSP5-8.5	2053	3.5922	3.2357	3.2458	4.2746	4.3232
SSP5-8.5	2054	3.1353	2.2793	2.2868	4.0620	4.3321
SSP5-8.5	2055	2.5636	2.1398	2.1467	2.7969	4.0023
SSP5-8.5	2056	2.9478	2.1302	2.1369	3.9268	4.9972
SSP5-8.5	2057	3.0790	2.1337	2.1428	4.3412	4.9109
SSP5-8.5	2058	4.5979	4.0530	4.0707	5.3760	5.3951
SSP5-8.5	2059	4.0905	3.6038	3.6123	4.3347	5.3718
SSP5-8.5	2060	3.1260	2.1993	2.2576	3.3018	5.3036
SSP5-8.5	2061	3.4921	3.1359	3.1445	3.3147	4.3547
SSP5-8.5	2062	1.9194	0.9857	0.9904	2.8641	3.0799
SSP5-8.5	2063	3.6494	3.0419	3.0469	4.3439	5.3047
SSP5-8.5	2064	2.7321	2.2684	2.2787	2.8390	4.8071
SSP5-8.5	2065	4.2974	4.0492	4.0636	4.2076	5.9019
SSP5-8.5	2066	4.7368	4.3185	4.3250	5.3780	5.3935
SSP5-8.5	2067	3.9719	3.2987	3.3032	5.2855	5.3795

Continue on the next page

Scenario	Year	Average	Q5	Q25	Q75	Q95
SSP5-8.5	2068	4.5765	4.2338	4.2481	5.3912	5.4160
SSP5-8.5	2069	4.8464	4.3349	4.3392	5.3965	5.4079
SSP5-8.5	2070	3.9077	3.2963	3.3013	4.3290	5.3796
SSP5-8.5	2071	4.3704	4.2048	4.2184	4.2791	5.3867
SSP5-8.5	2072	4.6800	4.3242	4.3283	5.3853	5.4008
SSP5-8.5	2073	4.6787	4.2914	4.3066	5.4166	5.4396
SSP5-8.5	2074	4.9974	4.3384	4.3433	5.4082	6.4750
SSP5-8.5	2075	4.2960	3.3013	3.3102	5.3793	5.4042
SSP5-8.5	2076	5.0029	4.2465	4.2647	5.4281	6.4709
SSP5-8.5	2077	5.5044	5.3813	5.4002	5.4221	6.4602
SSP5-8.5	2078	5.5633	5.3925	5.4080	5.4271	6.4910
SSP5-8.5	2079	5.6694	5.4019	5.4149	5.4510	6.5195
SSP5-8.5	2080	5.9216	5.4245	5.4387	5.4658	9.1036
SSP5-8.5	2081	5.9636	5.4309	5.4438	5.4687	9.6741
SSP5-8.5	2082	5.9006	5.4249	5.4392	6.4768	7.2855
SSP5-8.5	2083	5.8837	5.4169	5.4324	6.5087	6.5958
SSP5-8.5	2084	5.6475	5.4140	5.4299	5.4531	6.5396
SSP5-8.5	2085	6.6014	6.4772	6.5088	6.5573	6.5965
SSP5-8.5	2086	5.8696	5.4336	5.4476	6.5567	6.6105
SSP5-8.5	2087	5.7143	5.4416	5.4554	5.4846	6.5883
SSP5-8.5	2088	5.6137	5.4183	5.4302	5.4563	6.5405
SSP5-8.5	2089	5.7351	5.4126	5.4299	5.4559	8.1950
SSP5-8.5	2090	5.6012	5.4208	5.4348	5.4576	7.1831
SSP5-8.5	2091	5.6375	5.4023	5.4172	5.4474	6.5615
SSP5-8.5	2092	6.2508	5.4084	5.4260	6.5044	9.5953
SSP5-8.5	2093	6.8316	6.4636	6.4982	6.5677	8.4437
SSP5-8.5	2094	6.3014	5.4415	5.4640	6.5771	9.9585
SSP5-8.5	2095	6.6097	5.4593	5.4786	6.6875	10.6340
SSP5-8.5	2096	6.5291	5.4448	5.4626	6.7016	10.6853
SSP5-8.5	2097	7.4708	6.5027	6.5502	7.0981	11.3435
SSP5-8.5	2098	7.5768	6.5410	6.5957	7.3284	11.9792
SSP5-8.5	2099	7.1381	6.6317	6.6954	7.1297	8.9955
