# Supplementary material for: In hot water: Uncertainties in projecting marine heatwaves impacts on seagrass meadows
Source: PLoS One. 2024 Nov 27;19(11):e0298853. doi: 10.1371/journal.pone.0298853 (PMC11602073; doi:10.1371/journal.pone.0298853)
Supplement: Attachment — Submitted filename: Response to reviewers.pdf [file pone.0298853.s025.pdf]

Journal Requirements:

When submitting your revision, we need you to address these additional requirements.

1. Please ensure that your manuscript meets PLOS ONE's style requirements, including those for file naming.

The PLOS ONE style templates can be found at:

https://journals.plos.org/plosone/s/file?id=wjVg/PLOSOne_formatting_sample_main_body.pdf and,

https://journals.plos.org/plosone/s/file?id=ba62/PLOSOne_formatting_sample_title_authors_affiliations.pdf

2. Please note that PLOS ONE has specific guidelines on code sharing for submissions in which author-generated code underpins the findings in the manuscript. In these cases, all author-generated code must be made available without restrictions upon publication of the work. Please review our guidelines at https://journals.plos.org/plosone/s/materials-and-software-sharing#loc-sharing-code and ensure that your code is shared in a way that follows best practice and facilitates reproducibility and reuse.

3. We note that the grant information you provided in the ‘Funding Information’ and ‘Financial Disclosure’ sections do not match. When you resubmit, please ensure that you provide the correct grant numbers for the awards you received for your study in the ‘Funding Information’ section.

4. Thank you for stating the following financial disclosure: "This work was supported by the Centre of Data Science (CDS) and by QUT South American Scholarships." Please state what role the funders took in the study. If the funders had no role, please state: "The funders had no role in study design, data collection and analysis, decision to publish, or preparation of the manuscript." If this statement is not correct you must amend it as needed. Please include this amended Role of Funder statement in your cover letter; we will change the online submission form on your behalf.

5. Thank you for stating the following in the Acknowledgments Section of your manuscript: ' The authors also thank the Queensland University of Technology (QUT) for the financial support received through the South American Scholarship." We note that you have provided funding information that is not currently declared in your Funding Statement. However, funding information should not appear in the Acknowledgments section or other areas of your manuscript. We will only publish funding information present in the Funding Statement section of the online submission form. Please remove any funding-related text from the manuscript and let us know how you would like to update your Funding Statement. Currently, your Funding Statement reads as follows: "This work was supported by the Centre of Data Science (CDS) and by QUT South American Scholarships." Please include your amended statements within your cover letter; we will change the online submission form on your behalf.

6. We note that Figure 1 in your submission contain [map/satellite] images which may be copyrighted. All PLOS content is published under the Creative Commons Attribution License (CC BY 4.0), which means that the manuscript, images, and Supporting Information files will be freely available online, and any third party is permitted to access, download, copy, distribute, and use these materials in any way, even commercially, with proper attribution. For these reasons, we cannot publish previously copyrighted maps or satellite images created using proprietary data, such as Google software (Google Maps, Street View, and Earth). For more information, see our copyright guidelines:

http://journals.plos.org/plosone/s/licenses-and-copyright.

We require you to either (1) present written permission from the copyright holder to publish these figures specifically under the CC BY 4.0 license, or (2) remove the figures from your submission:

a. You may seek permission from the original copyright holder of Figure 1 to publish the content specifically under the CC BY 4.0 license. We recommend that you contact the original copyright holder with the Content Permission Form (http://journals.plos.org/plosone/s/file?id=7c09/content-permission-form.pdf) and the following text: "I request permission for the open-access journal PLOS ONE to publish XXX under the Creative Commons Attribution License (CCAL) CC BY 4.0 (http://creativecommons.org/licenses/by/4.0/). Please be aware that this license allows unrestricted use and distribution, even commercially, by third parties. Please reply and provide explicit written permission to publish XXX under a CC BY license and complete the attached form."

Please upload the completed Content Permission Form or other proof of granted permissions as an ""Other"" file with your submission. In the figure caption of the copyrighted figure, please include the following text: "Reprinted from [ref] under a CC BY license, with permission from [name of publisher], original copyright [original Copyright year]."

b. If you are unable to obtain permission from the original copyright holder to publish these figures under the CC BY 4.0 license or if the copyright holder's requirements are incompatible with the CC BY 4.0 license, please either i) remove the figure or ii) supply a replacement figure that complies with the CC BY 4.0 license. Please check copyright information on all replacement figures and update the figure caption with source information. If applicable, please specify in the figure caption text when a figure is similar but not identical to the original image and is therefore for illustrative purposes only. The following resources for replacing copyrighted map figures may be helpful:

USGS National Map Viewer (public domain):

http://viewer.nationalmap.gov/viewer/

The Gateway to Astronaut Photography of Earth (public domain):

http://eol.jsc.nasa.gov/sseop/clickmap/

Maps at the CIA (public domain):

https://www.cia.gov/library/publications/the-worldfactbook/index.html and

https://www.cia.gov/library/publications/cia-maps-publications/index.html

NASA Earth Observatory (public domain):

http://earthobservatory.nasa.gov/

Landsat:

http://landsat.visibleearth.nasa.gov/

USGS EROS (Earth Resources Observatory and Science (EROS) Center) (public domain):

http://eros.usgs.gov/#

Natural Earth (public domain):

http://www.naturalearthdata.com/

7. We notice that your supplementary tables are included in the manuscript file. Please remove them and upload them with the file type 'Supporting Information'. Please ensure that each Supporting Information file has a legend listed in the manuscript after the references list.

Authors Response:

Thank you for your detailed feedback on our manuscript submission. We have addressed the points as follows:

1. We have ensured that our manuscript meets PLOS ONE's style requirements, including proper file naming. We have utilised the provided PLOS ONE style templates for the main body and title, authors, and affiliations sections.

2. We have amended the financial disclosure statement to include the role of the funders. The revised statement reads: "The funders had no role in study design, data collection and analysis, decision to publish, or manuscript preparation." We have also removed the funding-related text from the Acknowledgments section of our manuscript. The updated Funding Statement now reads: "This work was supported by the Centre of Data Science (CDS) and by QUT South American Scholarships." This amended statement was included in the online submission form update cover letter.

3. Figure 1 Copyright Issue:
    - **World Heritage Areas of Queensland:** The data for World Heritage Areas of Queensland was obtained from the Queensland Government Spatial Catalogue, available at

      https://qldspatial.information.qld.gov.au/catalogue/custom/detail.page?fid={A49A0B80-277C-40DC-AFB5-D95E1EF9959B}

      This dataset is provided under the Queensland Government's open data initiative, which supports the distribution and use of its spatial data under the Creative Commons Attribution 4.0 International (CC BY 4.0) license.
    - **State controlled roads – Queensland:** The data for State Controlled Roads in Queensland was sourced from the same Queensland Government Spatial Catalogue, accessible at

      https://qldspatial.information.qld.gov.au/catalogue/custom/detail.page?fid={6BD377F3-B007-437B-A523-D7B26C07F545}

      This dataset is also covered under the CC BY 4.0 license, allowing for free use and distribution with proper attribution.
    - **States and Territories - 2021 (Shapefile); Local Government Areas - 2021 (Shapefile), and Suburbs and Localities – 2021:**

      https://www.abs.gov.au/statistics/standards/australian-statistical-geography-standard-asgs-edition-3/jul2021-jun2026/access-and-downloads/digital-boundary-files#metadata-for-digital-boundary-files

      Reference: Australian Bureau of Statistics (Jul2021-Jun2026), Access and downloads, ABS Website, accessed 17 July 2024.

4. We have removed the supplementary tables from the manuscript file and uploaded them separately with the file type 'Supporting Information.' Each Supporting Information file now includes a legend listed in the manuscript after the references list.

Review Comments to the Author

Please use the space provided to explain your answers to the questions above. You may also include additional comments for the author, including concerns about dual publication, research ethics, or publication ethics. (Please upload your review as an attachment if it exceeds 20,000 characters).

Reviewer #1:

In my opinion, the methodology presented here is powerful and promising to support risk analysis associated to predictions for marine ecosystems under climate change. I thoroughly enjoyed reading this manuscript as it is exceptionally well-written and presents a robust framework. In addition to the few points below, I suggest condensing the Discussion section slightly, although I acknowledge this may pose a challenge given the numerous projected scenarios. Except for some minor points (below), in my opinion, I recommend this manuscript for publication in PLOS ONE.

Authors Response:

Thank you for your thorough review and valuable feedback on our manuscript. We are pleased to hear that you found our methodology promising and the manuscript well-written. In response to your suggestion, we have made slight changes to the Discussion section for enhanced clarity.

Lines 741-744

Discussion:

We have rewritten "Conversely, the SSP3-7.0 and SSP5-8.5 scenarios, characterized by more pessimistic projections, reveal a marked decline in high shoot density ratios. SSP3-7.0 depicts a world fragmented by renewed nationalism, while SSP5-8.5 foresees unfettered, rapid growth. This decline in shoot density ratios can likely be attributed to increased emissions and less sustainable practices, adversely affecting *Z. muelleri*. Although the value spread for these percentiles is initially broad, it narrows as the century progresses." as "Conversely, under the medium-high emissions and population growth scenario (SSP3-7.0), as well as the highest emissions pathway (SSP5-8.5), a notable decline in the high shoot density ratios of *Z. muelleri* is observed. Although the spread of values for these percentiles is initially broad, it narrows as the century progresses."

Lines 813-816

Discussion:

We have rewritten "This method effectively characterizes uncertainties, greatly enhancing the model's value in ecological forecasting." to "While current metrics effectively evaluate resilience across various scenarios, future research could benefit from examining additional metrics to enhance our understanding of seagrass resilience, particularly under more challenging conditions."

Lines 349-363

Material and Methods:

We have moved the paragraph below to the "Measurement and Projections of Temperature Data" section in the Materials and Methods.

"Given the unpredictable nature of future human behavior, these scenarios present a spectrum of plausible climate futures, reflecting the outcomes of diverse policy choices. SSP1 advocates for

reduced emissions and a shift towards renewable energy, promoting a sustainable future path [115]. In contrast, SSP3, which has a higher radiative forcing, suggests a future marked by increased pollution and limited progress in health and education [116]. SSP5 envisions a heavy reliance on fossil fuels, resulting in significant increases in greenhouse gas emissions [117]. It is important that these RCP scenarios are not predictive and, therefore, do not carry associated probabilities [118]. Their primary purpose is to provide decision-makers with a range of potential outcomes derived from various plausible choices, thereby facilitating informed decision-making. As an example of how these projections can be used, consider the variability observed in the shoot density ratios of Z. muelleri under different SSP320 scenarios. This variability serves as a vital indicator, guiding strategic resource allocation in times of uncertainty and underscoring the importance of prioritising interventions in regions most likely to experience significant impacts."

Pg. 5, line 141: change to meadow

Authors Response:

We have revised "meadow" as suggested.

Line 189

"Our case study includes a *Zostera muelleri* seagrass meadow located at Pelican Banks, Gladstone Harbour (latitude: 23◦45′58′′S and longitude: 151◦18′30′′E), Australia (Fig 1)."

Pg. 8; lines 267-271- I wonder about the assumption of the 30 ºC threshold for shoot mortality, which seems to be only relying on data from 103. Wouldn't it be better to consider more studies with seagrasses from tropical areas (i.e. similar conditions to Gladstone)? Can the authors, please, comment on this point?

Authors Response:

Our thresholds were informed by studies on Zostera muelleri conducted in regions such as Cockle Bay and Picnic Bay on Magnetic Island, and Moreton Bay near the northern Great Barrier Reef — areas close to Gladstone.

References:

Collier, C. J., Ow, Y. X., Langlois, L., Uthicke, S., Johansson, C. L., O'Brien, K. R., ... & Adams, M. P. (2017). Optimum temperatures for net primary productivity of three tropical seagrass species. Frontiers in Plant Science, 8, 1446.

Collier, C. J., Uthicke, S., & Waycott, M. (2011). Thermal tolerance of two seagrass species at contrasting light levels: implications for future distribution in the Great Barrier Reef. Limnology and Oceanography, 56(6), 2200-2210.

We acknowledge the limitations of relying solely on these studies, particularly given the absence of direct data from Gladstone. To address this, we incorporated expert opinions when setting these parameters. We adjusted the threshold for seagrass shoot mortality from 33°C to 30°C based on literature reviews and climate projections that suggested daily averages below these thresholds. This decision considers that daily averages may not accurately reflect peak temperatures, which cause heat stress. Recognizing the limitation of basing this threshold on a single study, we agree that incorporating

data from additional studies, especially from tropical regions like Gladstone, would strengthen our model. Furthermore, exploring different thresholds could provide deeper insights; however, such analysis is beyond the scope of our current work. Our primary objective was to develop a methodology that integrates climate projections with dynamic Bayesian network models to assess impacts on marine ecosystems. The tool we developed has the capability to simulate and assess different thresholds, which can be explored in future work.

We have revised the methods section to include more clarification on how/why we chose this method. Additionally, in our discussion, we revisit this topic as a caveat to our study and place for future improvement and text is also included below.

Methods section

Lines 373-378

“The temperature thresholds used in the analysis were particularly informed by research conducted on *Zostera muelleri* in locations such as Cockle Bay and Picnic Bay on Magnetic Island, and Moreton Bay near the northern Great Barrier Reef—areas geographically proximate to Gladstone. Recognizing the limitations stemming from relying on data exclusively from these areas, especially given the absence of direct data from Gladstone itself, the thresholds were refined and validated through expert opinions.”

Discussion section

Lines 855-868

The paragraph “Furthermore, conducting sensitivity analyses would provide invaluable insights into the robustness of the model. Such analyses aim to identify the parameters with the most significant impact on overall risk estimates. Several methods could be employed for this, including calculating the partial derivatives of the risk estimate with respect to each parameter or utilizing advanced techniques like variance-based sensitivity analysis. These analyses would not only enhance the predictive reliability of the model but also guide future data collection and research efforts, ultimately contributing to a more robust and adaptable decision-support tool for marine ecosystem management. Lastly, the current study did not explore different thresholds for estimating temperature and heat stress state probabilities, akin to a scenario assessment. Future research could explore how variations in these thresholds might influence model outcomes. These identified gaps and limitations offer new opportunities for further investigation, potentially leading to more detailed and comprehensive findings in marine ecosystem management” has been rewritten as:

“Furthermore, conducting sensitivity analyses would provide invaluable insights into the robustness of the model, particularly in how thresholds are tested and validated. These analyses aim to identify the parameters that most significantly impact overall risk estimates. Several methods could be employed for this purpose, including calculating the partial derivatives of the risk estimate with respect to each parameter or utilizing advanced techniques like variance-based sensitivity analysis. Incorporating expert knowledge in these analyses is crucial, as it allows for the refinement of thresholds based on practical experience and insights that are not always captured by empirical data alone. This integration enhances the predictive reliability of the model and guides future data collection and research efforts, ultimately contributing to a more robust and adaptable decision-support tool for marine ecosystem management. Future work could also extend the research to capture how different heatwave frequencies and durations more comprehensively, and their variations over time might arise under different SSPs and their impact on resilience.

Additionally, the current study did not explore variations in thresholds for estimating temperature and heat stress state probabilities, akin to scenario assessments. Future research should consider how adjustments in these thresholds, informed by expert knowledge and sensitivity analysis outcomes, might influence model results. Such investigations would bridge identified gaps and limitations, offering new opportunities for more detailed and comprehensive findings in marine ecosystem management. These efforts are essential to ensure that the model remains adaptable and responsive to emerging data and expert consensus, thereby improving its utility as a decision-support tool."

Pg. 8, line 274: replace "ecosystem DBN" by either "framework" or "model" DBN

Authors Response:

Thank you for your suggestion to clarify the terminology in our manuscript. As per your recommendation, "ecosystem DBN" has been updated to "DBN model".

Lines 400-401

"The climate projections for the designated seagrass meadow include daily average temperature data. However, the DBN model operates on a monthly timescale."

Pg. 10, lines 336-338: I suppose there isn't previous data on shoot density, from the 1990's or early 2000's or before; if so, it would be preferable to assume this data as baseline. Please, comment.

Authors Response:

We do not have system-level data, including light, environmental conditions, and biological data from earlier time periods. Our study selected the baseline year (2022) for assessing shoot density in seagrasses as a specific climatological point rather than a mean over several years. While averaging multiple years could help mitigate the influence of exceptional annual conditions, using a single year as the baseline still provides a reference point across simulations, which can indeed be exceeded in certain scenarios. The choice of this baseline year takes into account that seagrasses exhibit seasonal fluctuations in their growth rates. By establishing a standard baseline, we were able to estimate the state probabilities for shoot density over the course of a year. Although we do not change the baseline throughout the study, we run samples through Monte Carlo simulations to account for possible variations in the scenarios. This baseline serves as a comparative reference for assessing changes and trends in seagrass density over time.

A recognized limitation of this approach, and an area for future research, is the potential variation in this baseline across extended time periods. The environmental and ecological conditions can change significantly over such a lengthy period, suggesting that the baseline itself might need to be adjusted to reflect these long-term changes. Future studies could explore different methodologies for adjusting the baseline over time to provide a more accurate and representative framework for evaluating seagrass density and health under changing climatic conditions.

We address this comment in the manuscript by providing more context in the section below.

Lines 468-469

"The baseline year for this study was set as 2022, based on the availability of environmental and biological ecosystem data. Significantly, 2022 marked the third consecutive year of good condition for Gladstone Harbour's seagrass meadows, following poor conditions from 2015 to 2018, making it an ideal reference for current and future assessments [133]. There is potential for significant environmental and ecological shifts over extended periods, underscoring the necessity to adjust the

baseline in future studies to reflect these long-term changes accurately and from a practical management standpoint".

Pg. 11 and 12, lines 375-377: reformulate the two sentences starting by "Post-integration of heat stress nodes".

Authors Response:

Thank you for pointing this out.

We have rewritten these sentences (lines 512-520) as:

"Post-integration of heat stress nodes, we aligned model results with [89] for baseline conditions. Post-integration of heat stress nodes, the output of the DBN was further subjected to qualitative evaluation using expert judgment [110]" to

"Prior to the integration of heat stress nodes, the model results were calibrated to align with baseline conditions as established by Wu *et al.* [100]. Additionally, the output of the DBN underwent a qualitative evaluation through expert judgment, as outlined in Kragt, M. E. [128]."

Pg. 13, line 443: change to there is; also change throughout the manuscript

Authors Response:

Thank you for pointing out the necessary grammatical corrections. I have updated "there's" to "there is" on page 17, line 604, and made consistent changes throughout the manuscript to reflect this correction.

"Fig 5a shows that in the SSP1-1.9 scenario, there is a potential to sustain higher population levels post-2060s."

Pg. 21- There's a repetition of the Conclusion section

Authors Response:

Thank you for pointing this out. We have rewritten the conclusion section as:

Lines 883-887

"This study provides significant insights into the resilience of *Z. muelleri* under diverse SSP scenarios, highlighting periods of high uncertainty that necessitate immediate action through intensified monitoring or further research to mitigate potential risks. Determining that SSP1-1.9 and SSP1-2.6 enable the best chance of recovery under future warming."

Figures- Improve the quality and definition of graphs and Figures

Authors Response:

In response to your comment, we have carefully reviewed each figure to ensure they meet the image quality standards required by the journal.

Reviewer #2:

This study aimed to develop a methodological framework that integrates ecosystem DBNs with climate model projections to assess the resilience of seagrass ecosystems and focuses on enhancing our understanding of seagrass responses to marine heat waves. The paper is well written, but there are some aspects that should be improved to clarify the understanding of the readers.

Authors Response:

Thank you for your detailed feedback and constructive comments. We appreciate your suggestions and have taken steps to address your concerns.

First of all, it is necessary to clarify how marine heatwaves are considered in this study. In the methods and results, the authors describe that they use SST data from CMIP6 models, but the number of marine heatwave events for the different scenarios and the duration are not assessed. This leaves me wondering whether you analysed marine heatwaves or simply SST variations over time.

Authors Response:

Regarding the consideration of marine heatwaves in our study, we have provided some clarify and we have also improved the text describing our methods in the manuscript.

Here's a breakdown of how the impact of MHWs on seagrass are modelled:

- Temperature and Heat Stress Nodes:
  The model includes specific nodes for temperature and heat stress. To capture the effect of marine heatwaves on seagrass, our study modelled variations in Sea Surface Temperature (SST) and their effects on the physiological function and mortality of seagrass using the "Temperature" and "Heat Stress" nodes, following the methodologies described by Hatum et al., 2024. Heat stress typically refers to conditions that are immediately detrimental to health or survival, whereas suboptimal temperatures can lead to less direct but nonetheless significant impacts, such as decreased growth rates.

- Projected Sea Surface Temperature:
  Temperature data is derived from climate model projections and is used to establish thresholds for seagrass resilience and mortality under heat stress conditions. The impact of marine heatwaves was integrated into our model through these nodes by utilizing daily SST projections through the year 2100. These projections considered different Shared Socioeconomic Pathways (SSPs), as discussed in the section "Measurement and Projections of Temperature Data."

- High-Frequency Data Integration:
  Alongside climate model projections, high-frequency SST data from a wave-monitoring buoy near Gladstone is used. This data, capturing temperature variations every 30 minutes, helps refine the understanding of daily temperature fluctuations critical for assessing thermal stress on seagrass.

- Threshold Establishment:
  Thresholds for temperature and heat stress are established using expert elicitation and peer-reviewed literature. In our study, we chose 33°C as the heat stress threshold based on literature, which serves as a critical benchmark for seagrass viability under heat stress conditions. However, given that the climate projections did not indicate temperatures reaching or exceeding 33°C, we opted to lower this threshold. This adjustment is based on the understanding that the projections provided are average daily temperatures, which likely underrepresent the maximum temperatures experienced over a 24-hour period due to daily variations. It is important to clarify that when referring to 'daily average' temperatures, we mean the average calculated for each individual model projection, not the mean across the ensemble of models. This approach assumes that the peak temperatures within any given day, which could potentially induce heat stress, are smoothed out in the daily averages provided by the models. As highlighted in the discussion section of our paper, the current study did not explore different thresholds for estimating temperature and heat stress state probabilities, similar to a scenario assessment. Recognizing this as a limitation, we suggest that future research could investigate how variations in these thresholds might affect model outcomes.

- Temporal and Spatial Modelling:
  The DBN model operates on a monthly timestep, integrating the daily and seasonal temperature variations to assess long-term impacts on seagrass resilience and recovery.

We have rewritten some sentences in the Material and Methods and Discussion to improve clarity:

Lines 245-255

“Building on the methodology introduced by Hatum et al. [104], our model captures the impact of marine heatwaves on seagrass resilience through two principal mechanisms. The first mechanism focuses on the optimal temperature range essential for plant growth, detailing the adverse effects on physiological status when temperatures deviate from this range. Such deviations can impair normal plant functions and disrupt the seed recruitment process, with younger plant stages being particularly vulnerable to elevated temperatures [105]. The second mechanism, heat stress, accounts for mortality or a reduction in shoot density resulting from temperatures that exceed the tolerance limits of the seagrasses. To model these effects, a temperature node is linked to physiological status and seed recruitment, while a heat stress node quantifies the loss in biomass, reflecting the direct impact of severe heat on seagrass mortality (Fig 2)”

Lines 325-336

“The downscaled CMIP6 climate models generate daily SST projections based on a range of SSPs, offering appropriate estimates for the temperatures experienced by intertidal seagrasses. Unlike their subtidal counterparts that remain continuously submerged, these intertidal species are periodically exposed to air, but the models estimate their overall temperature exposure using SSTs, not air temperature. Note that ‘daily average' temperatures refer to the average computed for each individual climate model projection rather than an ensemble mean across all models. This methodology tends to smooth out peak daily temperatures that might otherwise indicate potential heat stress. Therefore, to supplement the CMIP6 models' daily SST projections, we incorporated high-frequency SST data from a wave-monitoring buoy near Gladstone, accessible via the http://www.qld.gov.au/waves website.”

Lines 438-442

"Note that this assumption has limited influence on the outcomes of this specific one-month case study, rendering shorter intervals unnecessary for our current analysis. However, this approach could be modified to include shorter duration marine heatwave assessments at other sites or to investigate future deteriorations in the physiological conditions of this site ."

Lines 855-868

"Furthermore, conducting sensitivity analyses would provide invaluable insights into the robustness of the model, particularly in how thresholds are tested and validated. These analyses aim to identify the parameters that most significantly impact overall risk estimates. Several methods could be employed for this purpose, including calculating the partial derivatives of the risk estimate with respect to each parameter or utilizing advanced techniques like variance-based sensitivity analysis. Incorporating expert knowledge in these analyses is crucial, as it allows for the refinement of thresholds based on practical experience and insights that are not always captured by empirical data alone. This integration enhances the predictive reliability of the model and guides future data collection and research efforts, ultimately contributing to a more robust and adaptable decision-support tool for marine ecosystem management. Future work could also extend the research to capture how different heatwave frequencies and durations more comprehensively, and their variations over time might arise under different SSPs and their impact on resilience."

I have another question regarding mean sea level variations. Given that seagrasses are highly sensitive to changes in mean sea level, I wonder how this effect is accounted for in your model and in this study. Since several studies indicate that mean sea level is rising on the Australian coast and will continue to rise until 2100, it is crucial to clarify how this effect could impact seagrasses.

Authors Response:

Thank you for your inquiry regarding the impact of mean sea level variations on seagrasses. In this study, McWorter and Halloran's S2P3-R v2.0 downscaling simulations do not account for sea level rise (SLR). Based on predictions of approximately 1 meter of SLR by 2100 under the RCP8.5 scenario (IPCC, 2021), they concluded that such changes would not significantly affect their simulations; thus, computing resources were not dedicated to modeling this parameter.

Finally, it is crucial to clarify details about the model and its validation. I suggest adding a description of the model as supplementary material and clarifying how the model was validated.

Authors Response:

In response to the model validation, it is important to note that the model for Gladstone was previously validated by Wu et al., 2018. Additionally, we conducted a qualitative validation based on expert elicitations involving specialists in seagrass and climate modelling. These steps reinforce the reliability of the MSE values used in our assessments.

Lines 507-520

"The validation of the DBN model, as conducted by Wu et al. [100], involved a comparison of its predicted probabilities with those from external datasets not utilized during its training phase. These external datasets were derived from studies focused on the effects of light deprivation on seagrass, specifically simulating the impacts of dredging. Wu et al. [100] reported that the mean-squared error

(MSE) between the model's predicted-state probabilities and the observed values was in the range of 0.01 to 0.05. Prior to the integration of heat stress nodes, the model results were calibrated to align with baseline conditions as established by Wu et al. [100]. Subsequent to this calibration, the output from the DBN was subjected to qualitative evaluation through expert judgment, as detailed in Kragt, M. E. [128]. Additionally, the simulations of the downscaling climate models were validated using sea surface temperature data from satellite and mooring observations. Halloran et al. [108] identified a slight temperature bias, which may be attributed to the model operating as a one-dimensional vertical simulation that does not account for horizontal processes."

Specific comments

(139) Study area: Include more information.

What about tides, waves, sea levels and other factors (e.g. human induced) affecting seagrasses evolution? How study area seagrasses have evolved over time? Are there evidences that marine heatwaves affected its evolution? If yes, report the episodes.

Authors Response:

Thank you for your comment. We have addressed your suggestions by including additional information about the study area in the Materials and Methods section, specifically within the Gladstone Harbour case study.

Lines 200-220

"Seagrass evolution in the Port of Gladstone is influenced by a multitude of environmental and anthropogenic factors, including the local tidal and wave regimes. The area experiences variations between low-wave spring tides and high-wave neap tides, which play significant roles in the dispersal patterns of seagrass propagules [87, 88]. Frequent natural disturbances such as tropical storms, grazing by dugongs and turtles, and bioturbation enhance local clonal diversity by creating gaps that seeds colonize, preventing monopolization by competitively superior genotypes [89, 90]. Additionally, anthropogenic disturbances like dredging, land reclamation, and nutrient enrichment often increase sexual reproduction and genotypic diversity in seagrass meadows [91, 92]. High genotypic diversity is crucial for resilience to disturbances [93]. Genetic surveys reveal significant structuring among sites, influenced by the area's hydrodynamic complexity, resulting in varying connectivity and isolation [94, 95].

Projections indicate that sea-surface temperatures in the Great Barrier Reef could rise by 1°C to 3°C by 2100, with summer water temperatures potentially exceeding 33°C, well above the current distributional range of *Zostera muelleri* [96]. Seagrass populations in Gladstone have shown varying tolerance to heat stress. For instance, *Z. muelleri* from Gladstone exhibited high sensitivity to 33°C, suffering almost complete mortality after four weeks, whereas populations from Midge Point showed greater heat tolerance, maintaining positive productivity at 35°C and increasing thermal optima to 37.3°C [97]. Despite declines, such as those in southeast Queensland, Z. muelleri meadows often recover within three years[98, 99]."

Fig. 1 – Provide more details of the study area, including pictures of the seagrasses. Include labels with locations referred in the text: Gladstone Harbour; Pelican Banks; Great Barrier ReefWorld Heritage Area (GBR); Australia.

Authors Response:

We have updated Figure 1 to include more detailed information about the study area.

(line 152) Seagrass Dynamic Bayesian Network model

Consider including a significant portion of this information as supplementary material. For example, lines 153 to 165 contain a description of the model rather than a description of the methods.

Consider shortening fig. 2 caption. Journal recommendations state: avoid lengthy descriptions of methods.

Authors Response:

As suggested, we have shortened the caption of Figure 2 and relocated a portion of its text to Figure S1 in the Supporting Information. Additionally, we have moved lines 153 to 165 to the Supporting Information section (S1) to provide a more detailed description of the model there.

"Fig 2. Dynamic Bayesian Network Representation. Ovals represent nodes, and arrows indicate causal parent-child relationships, with the absence of a link suggesting conditional independence. Subnetworks are shown in rounded rectangles [77]. This representation is adapted from [100] and developed using GeNIe."

Supporting Information:

"S1 Fig. Seagrass Dynamic Bayesian Network. A BN is a class of graphical models representing the probabilistic relationships among a set of variables (or nodes) with directed arcs between them. These variables form a directed acyclic graph (DAG), and each node in the DAG has a state that varies depending on the states of other nodes [141]. Information about those states is transmitted throughout the DAG, and as a result, inferences may be made by adding new data or evidence to the network. While BNs are suitable approaches for inferring static processes, the DBN, a BN extension in which nodes represent variables at specific time slices, may be used to model temporally varying processes in which the state of a variable may change over time [142]. When adding a dynamic component to a BN and creating a DBN, the characteristic processes of complex systems, including cumulative effects and feedback processes, can be captured. The DBN approach allows us to predict the resilience of a system given the temporal dynamics of the components of the ecosystem and their interactions with natural and anthropogenic stressors [100, 106].

The overall DBN network structure is depicted with ovals representing factors (or nodes) and arrows indicating causal parent-child relationships. In this structure, a parent node (e.g., Meadow Type) exerts an influence on a child node (e.g., Location Type). Conversely, the absence of a link between nodes signifies conditional independence. Rounded rectangles denote subnetworks [77]. Note the presence of complex interdependencies and feedback loops in the seagrass ecosystem. Node colors denote different categories: site condition (white), recovery (purple), resistance (green), environmental (blue), and population (yellow). The following symbology is used in this figure: A node with a check mark: A node is ticked when inference has been successfully executed. A curved arrow back onto itself: Denotes a link to the node's next time slice (i.e., t + 1). A double-headed arrow: Indicates that arcs are heading both ways between two subnetworks. For example, a double-headed arrow between a node N and a subnetwork S means that there is at least one node in S that depends on N and that there is at least one node in S that influences N. An arrow labelled with a [1]: Indicates a connection to a subsequent time slice (t + 1)."

(213-216) Provide a detailed explanation of how you downloaded the CMIP6 data and describe in detail how you downscaled the data to a 10 km resolution.

Authors Response:

We have clarified this in three relevant areas, the main one being the Material and Methods, Section 'Measurement and Projections of Temperature' as follows:

Lines 295-305

"For accurate local-scale projections, particularly in the shallow coastal waters inhabited by seagrass, the semi-dynamic downscaling technique from Halloran et al. [106] was utilised to refine five CMIP6 models (MRI-ESM2-0 [110], EC-Earth3-Veg [111], UKESM1-0-LL [112], CNRM-ESM2-1 [113], IPSL-ESM2-0 [114]) to a 10 km resolution across four SSPs. This 1-D, semi-dynamic downscaling method inputs atmospheric variables (winds, downwelling shortwave and longwave radiation, pressure, humidity, air temperature) while accounting for local bathymetry and tidal conditions to perform a vertical water column calculation outputting SSTs [109, 115, 116]. The projections employ an ensemble mean, calculated as the average of all climate projections, with each projection being referred to as an ensemble member."

In addition, we have clarified in the Material and Methods, Section 'Measurement and Projections of Temperature':

Lines 325-336

"The downscaled CMIP6 climate models generate daily SST projections based on a range of SSPs, offering appropriate estimates for the temperatures experienced by intertidal seagrasses. Unlike their subtidal counterparts that remain continuously submerged, these intertidal species are periodically exposed to air, but the models estimate 312 their overall temperature exposure using SSTs, not air temperature, even though they are calculated from air temperature [109]. Note that 'daily average' temperatures refer to the average computed for each individual climate model projection rather than an ensemble mean across all models. This methodology tends to smooth out peak daily temperatures that might otherwise indicate potential heat stress. Therefore, to supplement the CMIP6 models' daily SST projections, we incorporated high-frequency SST data from a wave-monitoring buoy near Gladstone, accessible via the Coastal Data System - Waves website http://www.qld.gov.au/waves website."

In the Material and Methods, Section 'Modelling Temperature and Heat Stress':

Lines 438-442

"Note that this assumption has limited influence on the outcomes of this specific one-month case study, rendering shorter intervals unnecessary for our current analysis. However, this approach could be modified to include shorter duration MHW assessments at other sites or to investigate future deteriorations in the physiological conditions of this site."
