# Supplementary material for: In hot water: Uncertainties in projecting marine heatwaves impacts on seagrass meadows
Source: PLoS One. 2024 Nov 27;19(11):e0298853. doi: 10.1371/journal.pone.0298853 (PMC11602073; doi:10.1371/journal.pone.0298853)
Supplement: Attachment — Submitted filename: Response to reviewers - v2o.pdf [file pone.0298853.s026.pdf]

Editor Comment to the Authors:

Please review your reference list to ensure that it is complete and correct. If you have cited papers that have been retracted, please include the rationale for doing so in the manuscript text, or remove these references and replace them with relevant current references. Any changes to the reference list should be mentioned in the rebuttal letter that accompanies your revised manuscript. If you need to cite a retracted article, indicate the article's retracted status in the References list and also include a citation and full reference for the retraction notice.

Author's Response:

Reviewer #1 confirmed that all comments and suggestions were fully addressed, and the manuscript is ready for submission.

Reviewer #2's comments were addressed in detail:

1. The statement regarding "low-wave spring tides and high-wave neap tides" was corrected to accurately reflect that more energetic waves typically occur during high tides. The text was revised accordingly (lines 201-203).
2. We provided specific links to access the CMIP6 datasets, including the Centre for Environmental Data Analysis (CEDA) and downscaling details available on Zenodo (lines 293-298).
3. We added necessary links within the manuscript to direct readers to the supplementary material where relevant.

The rebuttal letter accompanying the revised manuscript reflects all changes made to the manuscript, including updates to the reference list.

Review Comments to the Authors:

Reviewer #1:

My comments and suggestions were fully addressed by the authors, as well as the comments from the other reviewer. Therefore, in my opinion, the paper is now ready for submission in PLOS ONE.

Author's Response:

Thank you very much for your thorough review and for acknowledging that our responses have fully addressed the comments and suggestions. We appreciate your time and effort in reviewing our manuscript and are grateful for your positive feedback.

Reviewer #2:

The paper has been modified in response to my comments and suggestions. It can be accepted, but first you need to respond to the following comments:

Author's Response:

Thank you for your review and your positive feedback. We appreciate your acknowledgment of the modifications made in response to your previous comments and suggestions. We have addressed the points you raised in detail below.

- [200] "... low-wave spring tides and high-wave neap tides,..." - Check that it's true. It is most common for more energetic waves to occur during high tides.

Author's Response:

Thank you for bringing this to our attention. Upon review, we have confirmed that the statement was incorrect. We have corrected the text to reflect that more energetic waves are typically associated with high tides.

"The area experiences variations between low-wave spring tides and high-wave neap tides, which play significant roles in the dispersal patterns of seagrass propagules [88, 89]." Has been rewritten as "The area experiences variations in wave energy, with more energetic waves typically occurring during high tides, which play significant roles in the dispersal patterns of seagrass propagules [88, 89]." (lines 201-203)

- [263] Be more specific. Provide the link of the platform to access the CMIP6 datasets

Author's Response:

Thank you for your suggestion. We have added the specific links for accessing the CMIP6 datasets.

"The CMIP6 datasets used to inform these scenarios were accessed through the Centre for Environmental Data Analysis (CEDA). Additionally, downscaling details are available via the Zenodo repository, as outlined in Halloran et al. [109]. The CMIP6 data can be accessed at https://catalogue.ceda.ac.uk, and the downscaling information is available at Zenodo DOI: 10.5281/zenodo.4147559." (lines 293-298)

- There is no link from the article to the supplementary material. It is important to direct the reader to the supplementary material when necessary, otherwise the supplementary material can be considered unnecessary.

Author's Response:

Thank you for pointing this out. We have reviewed the manuscript to ensure that all supplementary materials, including Supporting Figures (S Figs.) and Supporting Tables (S Tables), are adequately referenced in the text. We have identified and corrected instances where S Tables were not previously linked. Specifically, we have added references to these tables within the manuscript to ensure that readers are directed to the supplementary material whenever necessary as follows:

S1-16 -Table: line 470

"The first is a 90% interval, which spans from the 95th to the 5th percentiles, and the second is a 50% interval, ranging from the 75th to the 25th percentiles (S1 Table - S16 Table)."
